# Structural bases of inhibitory mechanism of Ca_V_1.2 channel inhibitors

**DOI:** 10.1038/s41467-024-47116-8

**Published:** 2024-03-30

**Authors:** Yiqing Wei, Zhuoya Yu, Lili Wang, Xiaojing Li, Na Li, Qinru Bai, Yuhang Wang, Renjie Li, Yufei Meng, Hao Xu, Xianping Wang, Yanli Dong, Zhuo Huang, Xuejun Cai Zhang, Yan Zhao

**Affiliations:** 1 Key Laboratory of Biomacromolecules (CAS), National Laboratory of Biomacromolecules, CAS Center for Excellence in Biomacromolecules, Institute of Biophysics, Chinese Academy of Sciences, Beijing, 100101 China; 2 College of Life Sciences, University of Chinese Academy of Sciences, Beijing, 100049 China; 3 State Key Laboratory of Natural and Biomimetic Drugs, Department of Molecular and Cellular Pharmacology, School of Pharmaceutical Sciences, Peking University Health Science Center, Beijing, 100191 China; 4 Heart Center and Beijing Key Laboratory of Hypertension, Beijing Chaoyang Hospital, Capital Medical University, Beijing, 100020 China; 5 Division of Life Sciences and Medicine, University of Science and Technology of China, Hefei, 230026 China

**Keywords:** Cryoelectron microscopy, Diseases of the nervous system, Ion channels, Biophysics

## Abstract

The voltage-gated calcium channel Ca_V_1.2 is essential for cardiac and vessel smooth muscle contractility and brain function. Accumulating evidence demonstrates that malfunctions of Ca_V_1.2 are involved in brain and heart diseases. Pharmacological inhibition of Ca_V_1.2 is therefore of therapeutic value. Here, we report cryo-EM structures of Ca_V_1.2 in the absence or presence of the antirheumatic drug tetrandrine or antihypertensive drug benidipine. Tetrandrine acts as a pore blocker in a pocket composed of S6^II^, S6^III^, and S6^IV^ helices and forms extensive hydrophobic interactions with Ca_V_1.2. Our structure elucidates that benidipine is located in the D_III_-D_IV_ fenestration site. Its hydrophobic sidechain, phenylpiperidine, is positioned at the exterior of the pore domain and cradled within a hydrophobic pocket formed by S5^DIII^, S6^DIII^, and S6^DIV^ helices, providing additional interactions to exert inhibitory effects on both L-type and T-type voltage gated calcium channels. These findings provide the structural foundation for the rational design and optimization of therapeutic inhibitors of voltage-gated calcium channels.

## Abstract (web summary)

CaV1.2 is crucial in cardiac, vascular and neuronal function, serving as a target for many drugs. Here, authors identify the binding site of herb-derived drug tetrandrine, and explore inhibitory mechanism of L/T-type selective DHP drug benidipine.

## Introduction

Voltage-gated calcium channels can be classified into high voltage activated calcium channels (HVAs) and low voltage activated calcium channels (LVAs)^1,2^. HVA (Ca_V_1 and Ca_V_2) contains pore-forming subunit α1, auxiliary subunits α2δ and β, whereas LVA (Ca_V_3) contains only α1. Ca_V_1 channels are defined as L-type due to their long-lasting current^3^. L-type Ca_V_1.2 plays an essential role in cardiac and smooth muscle contractility, neuroendocrine regulation, and multiple other processes^4–6^. It is expressed in the brain, cardiomyocytes, sinoatrial node, vascular system, pancreatic islets, arsenal medulla, and intestinal/bladder smooth muscle. Ca_V_1.2 is predominant in contraction in atrial, ventricular, and vascular excitable tissues as it triggers the release of calcium from the sarcoplasmic reticulum terminal cistern for actin-myosin interaction and thus is the uppermost target for the treatment of hypertension and one of the therapeutic targets of arrhythmia^4^. Meanwhile, approximately 90% of the L-type calcium channels in the brain are Ca_V_1.2 (refs. ^7–9^). It is highly expressed in hippocampal neurons, participates in long-term synaptic plasticity and is essential for the formation of spatial memory, fear memory, and emotional behaviors^9–14^. Its malfunction is related to schizophrenia and bipolar affective disorder^15^. Ca_V_1.2 also plays an essential role in early development, regulating chondrogenesis^16,17^. Inherited mutations of Ca_V_1.2 are associated with serious diseases, including cardiac and vessel diseases as well as neuronal disorders^18–20^.

The discovery of more drug binding patterns is one of the practical goals in structural biology. There are various marketed drugs targeting Ca_V_1.2. In previous studies, the structures of L-type calcium channels bound with dihydropyridine (commercial name: nifedipine), phenylamine (verapamil), benzothiazine (diltiazem), and diphenylmethyl piperazine (cinnarizine) were resolved^21,22^. However, there are still unidentified inhibition mechanisms of clinical drugs remaining to be clarified. For instance, tetrandrine is a dibenzyl isoquinoline alkaloid and functions as a voltage gated calcium channel antagonist^23–25^. Similar to the well-known artemisinin, tetrandrine is an effective ingredient extracted from traditional Chinese medicine and is attracting more attention due to its diverse pharmacological effects. It is currently used in the clinic for anti-rheumatism and analgesia, as well as for the treatment of lung cancer and silicosis, and shows an effect of lowering blood pressure^26–30^. Analyzing the binding mode between Ca_V_1.2 and tetrandrine can expand our understanding of ion channel inhibitors and provide structural bases for further modification and optimization of such drugs. However, different sidechain modifications of dihydropyridine may cause different pharmacological effects and varied affinity toward ion channels. For example, in guinea pig ventricular cells, the IC_50_ values of benidipine and nifedipine are at least 10 times different, providing a reference for affinity optimization of dihydropyridine or other drugs. To explain these phenomena, further analyses of more dihydropyridine drugs are still desirable. Moreover, many pathogenic mutations cause distortion of the electrophysiological properties of Ca_V_1.2. As pathogenic mutation sites are usually not strictly conserved in L-type calcium channels, the lack of Ca_V_1.2 structure limits the understanding of the pathogenic mechanism of these mutations.

In this study, we determine the near-atomic resolution cryo-EM structure of apo Ca_V_1.2 and analyzed the distribution pattern as well as potential impacts on the gating of a collection of pathogenic mutations in Ca_V_1.2. In addition, we solve the Ca_V_1.2 complex structures in the presence of tetrandrine or benidipine. Tetrandrine blocks the pore of Ca_V_1.2, interacting with hydrophobic residues in the central cavity. The mutations N741W (Asn-to-Trp mutation at the position 741) and N1179W can significantly reduce the inhibition of tetrandrine, confirming that a large cavity space is needed for tetrandrine blocking. Benidipine is located in the fenestration between D_III_ and D_IV_. The hydrophobic sidechain of benidipine provides extra interactions with the channel subunit α1, supporting the inhibition of both L- and T-type voltage gated calcium channels. These results elucidate how the chemical groups of these inhibitors subtly remodeled their interactions with Ca_V_1.2.

## Results and discussion

### Architecture of the Ca_V_1.2 channel complex

To investigate the inhibitory mechanism of Ca_V_1.2-targeting drugs, we co-expressed the human Ca_V_1.2 with its physiological auxiliary subunits α2δ1 and β2b in HEK293 cells. The genes encoding these three subunits were subcloned into pEG-BacMam vector. We also carried out whole-cell patch clamp using HEK293T cells to characterize the functional properties of these constructs (Supplementary Fig. 1a). The subunits expressed with these constructs form a functional complex, showing an activation curve similar to those from previous studies^31,32^, and the half-activation voltage (V_1/2_) of the recombinant channel is +1.0 (±0.5) mV. Subsequent purification experiments resulted in a sharp and symmetrical SEC profile, indicating homogeneity of the sample of the channel complex. The SDS-PAGE gel confirmed that the purified sample contained all three subunits (Supplementary Fig. 1b, c). Next, we collected cryo-EM data using Titan2 Krios and carried out single-particle cryo-EM analysis. The three-dimensional cryo-EM maps of Ca_V_1.2 in the apo state (denoted as Ca_V_1.2^apo^), tetrandrine bound state (Ca_V_1.2^TET^), and benidipine bound state (Ca_V_1.2^BEN^) were determined at 3.5-Å, 3.4-Å, and 3.3-Å resolutions, respectively. These maps are rich in structural features, including densities for sidechains, N-glycans, and lipid molecules, and allowed us to reliably build atomic models of these Ca_V_1.2 complexes (Supplementary Figs. 2–3) (Supplementary Table 1).

**Fig. 1:**
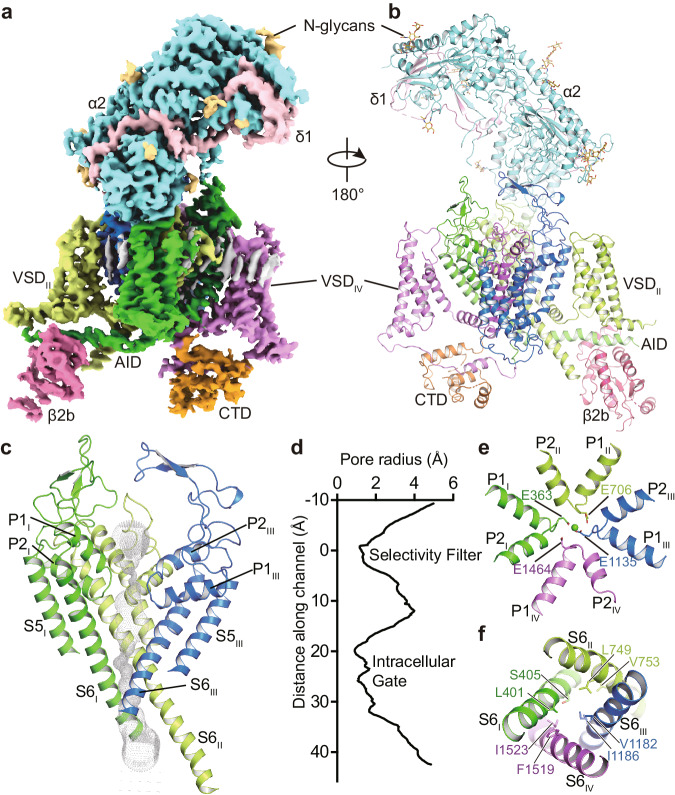
Architecture of the Ca_V_1.2 complex. **a**, **b** The overall EM density map and structure of the Ca_V_1.2 complex. The α2δ1 and β2b subunits, voltage-sensing domain (VSD), C-terminal domain (CTD), α-interacting domain (AID), and N-glycans were labeled. The α1 subunit is colored in deep green (Domain I, D_I_: A114-R211, F231-I447), light green (D_II_: W509-K778), deep blue (D_III_: F889-I943, F964-Y1197), mauve (D_IV_: K1198-S1321, E1350-T1357, S1372-E1484, P1494-D1533), and orange (CTD: W1534-P1574, C1582-S1593, N1607-K1638, K1647-P1655), respectively. The β2b (D222-R276, L297-W407), α2 (F27-D129, Q143-V224, K232-N540, Q554-K690, N698-T828, D849-P906), and δ1 (Q972-G1022, T1028-D1070) subunits are colored in magenta, turquoise, and pink, respectively. N-glycans are colored in yellow. **c** The ion permeation path in the pore domain. The selectivity filter and S5-S6 helices are shown in cartoon and viewed in parallel to the membrane plane. The ion conducting pathway was calculated by using the program HOLE and illustrated with gray dots. **d** Plot of pore radii for the Ca_V_1.2 complex. **e** The selectivity filter ring of four glutamate residues from the four domains of Ca_V_ channel were shown in sticks. A cation ion is shown as a green sphere. **f** The intracellular gate formed by four S6 helices viewed from the intracellular side. Hydrophobic residues are shown in sticks.

The structure of the Ca_V_1.2 channel complex is composed of a pore-forming α1 subunit and auxiliary subunits α2δ1 and β2b (Fig. 1a, b). Its α1 subunit consists of four repeat transmembrane domains (D_I_−D_IV_). Each domain contains six transmembrane helices (S1−S6) and forms the channel in a domain-swapped fashion. The S1−S4 helices constitute the voltage sensing domain (VSD) to detect changes in the cross-membrane electrostatic potential_._ Consistent with all known structures of voltage-gated channels, the S4 helix assumes a 3_10_-helical conformation. The S5−S6 regions of all four domains form the pore domain to facilitate and control the passage of ions (Fig. 1c, d). The selectivity filter (SF) is contributed by the re-entrant loops from each subunit that connect S5 and S6 and include P1 and P2 helices (Fig. 1e). In contrast to the conformational heterogeneity of the α-interaction domain (AID) found in Ca_V_1.1 (ref. ^33^), AID is well resolved in the current Ca_V_1.2 structure, with its N- and C-termini adjacent to the intracellular gate and VSD_II_, respectively (Fig. 1a). The selectivity filter contains four acidic residues (E363^DI^, E706^DII^, E1135^DIII^, and E1464^DIV^; i.e. the EEEE motif), one from each domain, producing a strongly negatively charged zone to attract cations and determine selectivity for Ca^2+^ ions^33^ (Fig. 1e). On the cytoplasmic side, the four S6 helices bundle together and act as a gate to control the access of ions into the pore (Fig. 1f). The pore diameter profile indicates that the current structure represents an inactivated state with a closed intracellular gate (Fig. 1d). Although all S4 helices in the four VSDs are in an ‘up’ conformation, the intracellular gate remains closed, suggesting that the structure of Ca_V_1.2 was captured in an inactivated state. In addition, the α2δ1 subunit was clearly resolved and sits on the extracellular side of Ca_V_1.2, interacting with the E149, D150, and D151 residues from the D_I_ repeat. The β2b subunit is composed of a GK domain and an SH3 domain and is associated with the cytosolic AID region of Ca_V_1.2 (Fig. 1). A lipid molecule was observed in the D_I_−D_II_ fenestration of Ca_V_1.2^apo^, consistent with other structure studies on Ca_V_1.2 (refs. ^34–36^).

The structure of Ca_V_1.2^apo^ was compared with that of Ca_V_1.1 (PDB ID: 5GJW)^33^, giving rise to an RMSD of 1.35 Å for 1772 C_α_-pairs. The overall structure, including VSD, SF, and extracellular loops (ECLs), is fairly superimposable (Supplementary Fig. 4a). In both structures, in the absence of applied membrane potential, all VSDs show an “up” conformation of the S4 helices and are thus in the “activated” state. However, some structural differences are observed between the structures of Ca_V_1.2^apo^ and Ca_V_1.1. For example, two π-bulges occur on the S6^DI^ and S6^DIII^ helices of Ca_V_1.2^apo^, whereas both helices assume a canonical α-helix conformation in Ca_V_1.1. The sidechains of residues on the intracellular segment, starting from F394^S6I^ and F1175^S6III^, undergo an approximate 90° rotation (Supplementary Fig. 4b). The residues forming the central cavity and the intracellular gate differs from that in Ca_V_1.1. In specific, the side chain of F394 and F1175 face the central cavity, causing a shrinkage of the inner radius of the pore domain (Supplementary Fig. 4c, d). In Ca_V_1.1, the intracellular gate is constituted by V329, L333, F656, A660, F1060, V1064 F1376, and I1380. With the local conformational shifts on the S6 helices in Ca_V_1.2, the intracellular gate is formed by L401, S405, L749, V753, V1182, I1186, F1519, and I1523 (Supplementary Fig. 4e). A previous study on TRPV6 channel suggested that the transition from an α-helix to a π-helix may prompt channel opening^37^. However, despite such transition being observed in Ca_V_1.2, its intracellular gate remains closed (Supplementary Fig. 4f).

### Pathogenic mutations of Ca_V_1.2

**Fig. 2:**
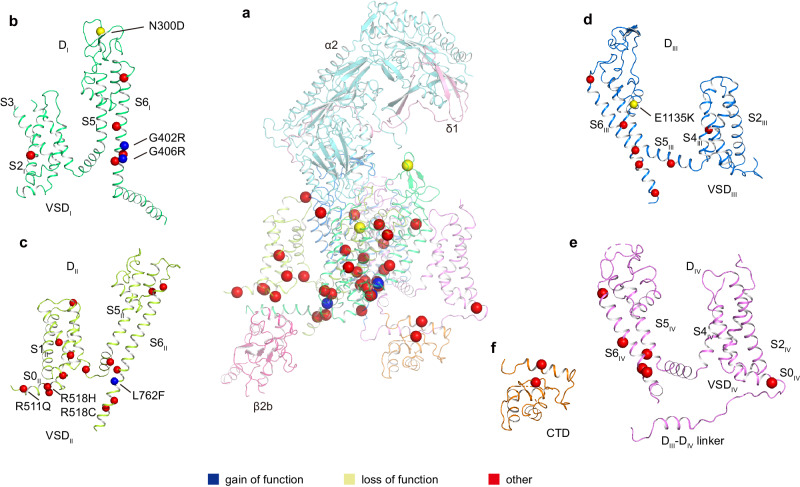
Molecular mapping of pathogenic mutations. **a**–**f** Disease related mutations are located on D_I_ (**b**), D_II_ (**c**), D_III_ (**d**), D_IV_ (**e**), and CTD (**f**). D_I_, D_II_, D_III_, D_IV_, and CTD of α1 subunit are colored in deep green, light green, deep blue, mauve, and orange, respectively. Subunits α2, δ1, and β2b are colored in turquoise, pink, and magenta, respectively. Mutation sites are marked with spheres and colored in blue (gain of function), yellow (loss of function), and red (other), respectively.

Dysfunction of Ca_V_1.2 usually causes heart and vessel diseases and neurological disorders^18–20^. At least 86 mutations of the Ca_V_1.2 α1 subunit have been identified in various cases of human diseases, including Timothy syndrome (TS), long QT syndrome 8 (LQTS), short QT syndrome (SQTS), Brugada syndrome (BrS), autism, and schizophrenia, among others (Supplementary data). Our current Ca_V_1.2 structure provides a template to map these disease-related mutations into the 3D structure. Among these mutants, 39 sites are distributed on the four VSDs, pore domain, and C-terminal domain (CTD) (Fig. 2). The VSD_II_ and pore domain harbor most of the pathogenic mutations. The remaining 47 mutation sites are mainly distributed on the N-terminus, cytosolic long linkers between different domains (mainly D_II_−D_III_), and the C-terminal tail, and they were not determined due to conformational heterogeneity.

Three gain-of-function (GOF) mutations have been identified and verified by electrophysiological experiments. G402S and G406R were found in Timothy syndrome cases, and they inhibit voltage-dependent inactivation of Ca_V_1.2 (refs. ^18,38^). Since all G406 substitutions to other amino acid residues, such as alanine, serine, and valine, impair the inactivation process, the function of G406 is proposed as irreplaceable^38^. In our Ca_V_1.2 structure, both G402 and G406 are located on the S6^DI^ helix and close to the intracellular gate (Supplementary Fig. 5b). We speculate that the absence of a sidechain in glycine at these two positions is essential for the conformational transition from the open state to the inactivated state, and replacement by a sidechain-containing residue at either of these two positions probably hinders the transition.

In a previous study, L762F associated with LQTS was found to result in a GOF defect with slower inactivation^39^. In the current Ca_V_1.2 structure, L762 on the S6^DII^ helix is located around the cytosolic membrane surface. Its sidechain points toward the AID motif and the S4-S5^DII^ linker helix (Supplementary Fig. 5c). We speculate that the mutation L762F results in steric hindrance with AID or S4-S5^DII^ helices, leading to a conformational change in these helices and affecting channel inactivation kinetics.

N300D is a loss-of-function (LOF) mutation and has been identified in Brugada syndrome patients^40^. This mutation leads to a decrease in the current density by impairing the expression level of Ca_V_1.2 in the membrane^40^. N300 is located in the extracellular loop between S5^DI^ and S6^DI^ (Supplementary Fig. 5a) and is exposed to the interface between the α1 and α2δ1 subunits, which is essential for trafficking of the Ca_V_1.2 complex^41,42^. N300D may thus affect traffic by disturbing the interaction with α2δ1. In addition, E1135K is another LOF mutation identified in patients with idiopathic QT prolongation, bradycardia, and autism spectrum disorder^43,44^. This mutation is positioned in the EEEE motif of the selectivity filter in the current Ca_V_1.2 structure (Fig. 1e and Supplementary Fig. 5d). Electrophysiological experiments demonstrated that the E1135K mutation makes Ca_V_1.2 a nonselective monovalent cation channel, in line with functional roles of the EEEE motif in determining ion selectivity^44,45^.

Previous studies have indicated that R511Q, R518C, and R518H are associated with LQTS and cardiac only Timothy syndrome^46,47^. In the current Ca_V_1.2 structure, R511 and R518 are located at a cytosolic amphiphilic helix N-terminal to S1^DII^ (denoted as S0^DII^). The S0^DII^ helix is rich in positive charged residues (^511^RFCRRKCRAAVK^522^) that electrostatically interact with the negatively charged C-terminus of AID (Supplementary Fig. 5e). Electrophysiological experiments demonstrated that pathogenic mutations associated with R511 and R518 result in slower inactivation^46,47^. We speculate that these mutations interfere with the interaction between S0^DII^ and AID and thus affect the conformation of AID relative to the pore domain, resulting in reduced inactivation. This speculation is consistent with the notion that AID is important for regulating channel inactivation^48,49^.

### Recognition of tetrandrine

**Fig. 3:**
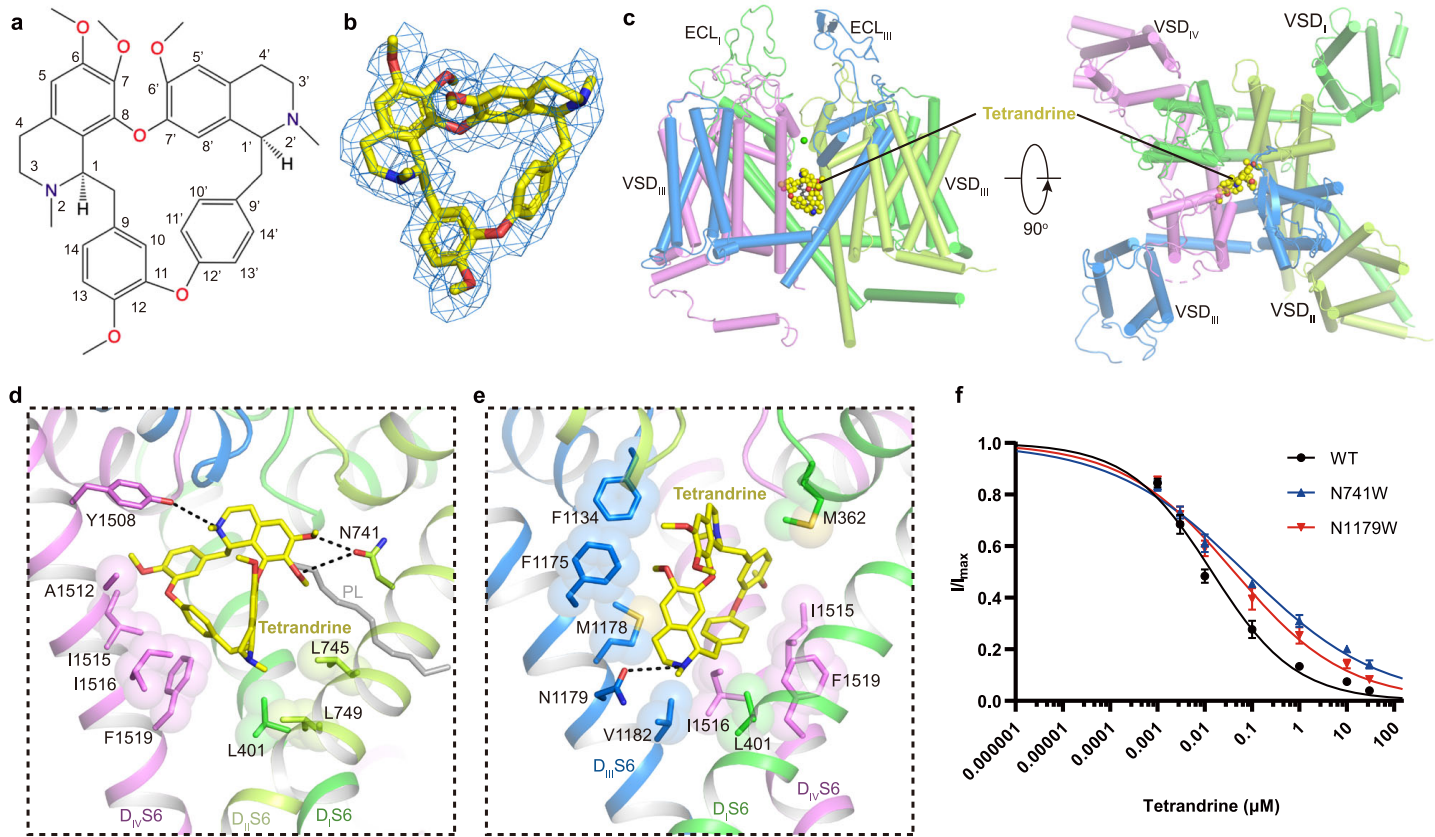
Structure basis for blockade of Ca_V_1.2 by tetrandrine. **a** Chemical structure of tetrandrine. **b** The cryo-EM density shown in blue mesh for tetrandrine in sticks. **c** The overall structure of the Ca_V_1.2^TET^ complex. The domains of Ca_V_1.2^TET^ are colored as D_I_ in deep green, D_II_ in light green, D_III_ in deep blue, and D_IV_ in mauve. The tetrandrine located in the pore domain is presented as yellow spheres. **d**, **e** Detailed binding sites for tetrandrine showing interactions between tetrandrine and Ca_V_1.2. The sidechains of key residues are displayed in sticks and the hydrophobic side chains are overlaid with transparent surfaces. Black dashed lines indicate potential hydrogen bonds. A lipid located within the fenestration site is shown in gray. **f** Dose-response curves of tetrandrine inhibition on Ca_V_1.2^WT^, Ca_V_1.2^N741W^, and Ca_V_1.2^N1179W^. Ca_V_1.2^N741W^ and Ca_V_1.2^N1179W^ shift the IC_50_ of tetrandrine on Ca_V_1.2 from 14.2 nM to 71.3 nM and 41.8 nM, respectively. Data in curves are represented as mean ± SEM (*n* = 10 biologically independent experiments). Source data are provided as a Source Data file.

Tetrandrine is a traditional Chinese clinical agent for autoimmune disorders, cardiovascular diseases, and hypertension. It is extracted from the root of *Stephania tetrandra* S Moore and inhibits both L-type and T-type Ca_V_ channels^25,50,51^. To explore the molecular basis how tetrandrine blocks the activity of Ca_V_1.2, tetrandrine (10 μM) was added during protein expression and purification. We determined the tetrandrine-bound Ca_V_1.2 complex (Ca_V_1.2^TET^) at 3.4-Å resolution (Supplementary Fig. 2). In our cryo-EM map, we found a well-resolved triangle-shaped density in the central cavity of the pore domain, which is well fitted with the tetrandrine molecule and positioned proximal to the selectivity filter (Fig. 3a–c). The two major moieties of the 6,7-dimethoxy-2-methyl-1-benzyl-isoquinoline ring are positioned close to the D_II_−D_III_ fenestration site and D_IV_, and they are stabilized by forming hydrophobic or Van Der Waals interactions with surrounding residues from the selectivity filter and S6 helices (Fig. 3d, e). In particular, the residues F1175^S6III^, M1178^S6III^, A1512^S6IV^, I1516^S6IV^, and F1519^S6IV^ play important roles in stabilizing tetrandrine. The 6,7-dimethoxy and 2/2’-N groups from the isoquinoline ring form hydrogen bonds with the N741^S6II^ and Y1508^S6IV^/N1179^S6III^ residues. In addition, in agreement with observations in drug-bound Ca_V_3.3 complex structures^52^, a lipid molecule penetrates through the fenestration site between D_I_ and D_II_ and participates in hydrophobic interactions with the tetrandrine molecule (Fig. 3d, e). Compared with other drug binding modes observed in the previously reported Ca_V_1.1 structures, we found that the tetrandrine binding mode is distinct from those of nifedipine (a DHP derivative), verapamil (PAA), diltiazem (BTZ), and cinnarizine (CIN) (Supplementary Fig. 6a). In particular, tetrandrine completely squeezes the binding site of BTZ (Supplementary Fig. 6b), consistent with previous experimental results showing that tetrandrine and diltiazem cannot simultaneously bind to L-type Ca_V_ channels^53^. To validate the binding site of tetrandrine, we designed two mutants, N741W and N1179W, with the potential to create steric clash between tetrandrine and the binding site. We determined the dose-response curves of tetrandrine for both wild-type and these two mutants. The curves for both mutants exhibited a rightward shift compared to that of wild-type Ca_V_1.2, indicating that these mutants reduce the sensitivity of these mutants to tetrandrine. The derived IC_50_ values for tetrandrine with the N741W and N1179W mutants are 71.3 nM and 41.8 nM, respectively, which are higher than that of wild-type (~14.2 nM) (Fig. 3), further confirming the binding site of tetrandrine.

### Antagonism of benidipine

1,4-Dihydropyridine derivatives (DHPs), such as nifedipine, selectively inhibit certain voltage gated calcium channels and are widely used clinically for the treatment of hypertension^54^. However, DHPs frequently cause side effects such as proteinuria, upon chronic use. The specific inhibition of L-type calcium channels is usually an advantage but is a defect in the kidney. The reason is that L-type channels are distributed in the kidney glomerulus afferent arteriole but are absent in the efferent arteriole. This inconsistent inhibition results in unbalanced local blood pressure, leading to kidney damage with proteinuria^55,56^. However, benidipine, another DHP derivative, can additionally inhibit T-type calcium channels, which are more abundant in efferent arterioles than in afferent arterioles, making blood pressure more balanced in different parts of the kidney. Compared with nifedipine and amlodipine, benidipine causes fewer adverse side effects, such as proteinuria and leg edema^55,57,58^; therefore, it is widely used in the clinic. Compared with nifedipine, benidipine replaces 8-nitrophenyl with 9-nitrophenyl and 3-carboxylic acid methyl ester with 3-(1-phenylmethyl-3-piperidinyl) ester. By supplementing benidipine when solubilizing the membrane and before preparing the cryo-EM sample, we obtained a 3.3-Å resolution benidipine-bound Ca_V_1.2 structure (Ca_V_1.2^BEN^) (Supplementary Fig. 2).

**Fig. 4:**
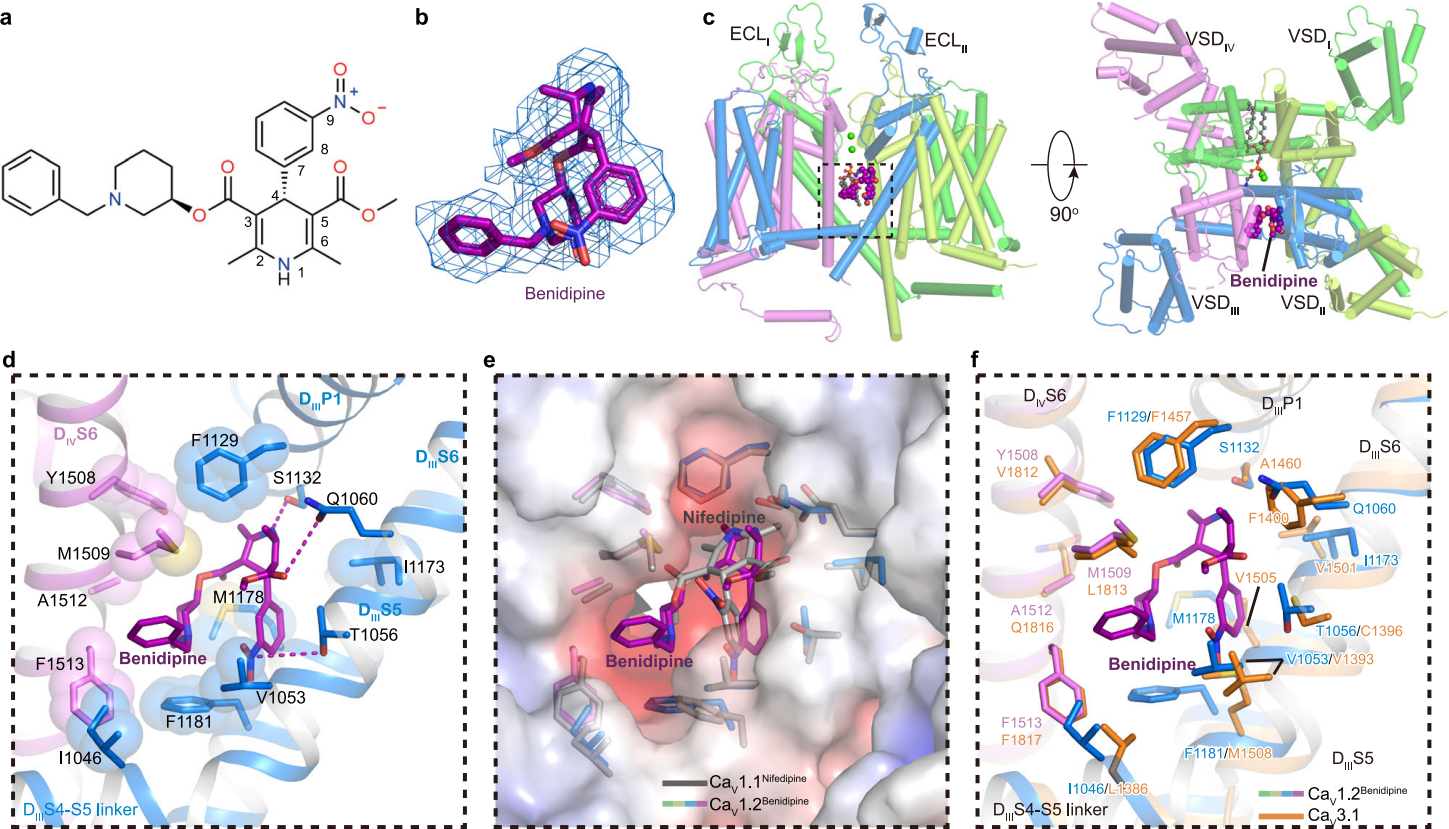
Structure basis for inhibition of Ca_V_1.2 by benidipine. **a** Chemical structure of benidipine. **b** The cryo-EM density shown in blue mesh for benidipine in sticks. **c** The overall structure of the Ca_V_1.2^BEN^ complex. The domains of Ca_V_1.2^BEN^ are colored as D_I_ in deep green, D_II_ in light green, D_III_ in deep blue, and D_IV_ in mauve. The tetrandrine is presented as violet spheres. Two cation ions are shown as green spheres. Phospholipid entering through fenestration is shown as gray sticks. **d** Detailed binding sites for benidipine showing interactions between benidipine and Ca_V_1.2. The sidechains of key residues are displayed in sticks and the hydrophobic side chains are overlaid with transparent surfaces. Black dashed lines indicate potential hydrogen bonds. **e** Comparison of the DHP ligands binding sites of Ca_V_1.2 with nifedipine bound Ca_V_1.1 structure (PDB ID: 6JP5) (colored in gray), overlaid with the electrostatic surface potential of Ca_V_1.2. **f** Comparison of the benidipine binding sites of Ca_V_1.2 with Ca_V_3.1 (PDB ID: 6KZO) (colored in orange).

The benidipine molecule penetrates into the D_III_−D_IV_ fenestration and corresponds to a ‘claw’ shape density in the map (Fig. 4a, b). Unlike the pore-blocker tetrandrine, benidipine does not directly block the pore but allosterically inhibits Ca_V_1.2. The main moiety of dihydropyridine is biased towards D_III_ (Fig. 4c). The nitrogen atom on the pyridine ring forms a potential hydrogen bond with S1132^P1^ (Fig. 4d). The nitrogen atom on 9-nitrophenyl and the oxygen atom on 5-dicarboxylic acid of the pyridine ring form hydrogen bonds with T1056^S5III^ and Q1060^S5III^, respectively (Fig. 4d). Whereas similar interactions with T935 and Q939 have been identified in the structure of the Ca_V_1.1 complex, benidipine also contains a phenylmethyl piperidinyl group, which is positioned in a hydrophobic pocket formed by residues I1046^S5III^, I1049^S5III^, V1053^S5III^, F1181^S6III^, M1509^S6IV^, and F1513^S6IV^, providing additional hydrophobic interactions to stabilize its binding, in line with the inhibitory IC_50_ of benidipine being approximately ten times smaller than that of nifedipine^59^ (Fig. 4d, e). Previous studies indicated that nifedipine specifically inhibits L-type Ca_V_ channels, showing no sensitivity to N- and T-type Ca_V_ channels^60–62^. In contrast, benidipine, in addition to L-type Ca_V_ channel, can also inhibit both N-type and T-type Ca_V_ channels, with IC_50_ values of approximately 35 μM and 11 μM, respectively^59,63,64^. To understand the molecular basis of how benidipine is recognized by Ca_V_3.1, we compared the benidipine-bound Ca_V_1.2 structure with that of Ca_V_3.1, and the result illustrates that most of the residues participating in benidipine binding are also conserved in Ca_V_3.1, such as V1053^S5III^, F1129^DIIIP1^, and F1513^S6IV^. Although I1046^S5III^, M1178^S6III^, F1181^S6III^, and M1509^S6IV^ in Ca_V_1.2 are substituted by L1386^S5III^, V1505^S6III^, M1508^S6III^, and L1813^S6IV^ in Ca_V_3.1, respectively, we speculate that these substitutions by similar residues are tolerable and that the relatively conserved binding pocket underlies the basis for how benidipine acts as an inhibitor in multiple voltage gated calcium channels (Fig. 4f). Moreover, two residues, T1056 and Q1060, play pivotal roles in the binding of DHP drugs to L-type Ca_V_ channels. However, these residues are not conserved in N-type and T-type Ca_V_. Residue T1056 of Ca_V_1.2 is substituted by Y1289 in Ca_V_2.2, and residue Q1060 is replaced by M1293 in Ca_V_2.2 and F1400 in Ca_V_3.1 (Supplementary Fig. 7a, b, Fig. 4f). Although benidipine does not inhibit mutations T1056Y, Q1060M, and Q1060F as potently as it does in WT Ca_V_1.2, it still retains the ability to block the current (Supplementary Fig. 7c). In contrast, previous studies demonstrated that these same substitutions at positions T1056 and Q1060 almost abolished the sensitivity of Ca_V_1.2 to the DHP drug R202-791 (ref. ^65^). The varied effects of these mutations on benidipine and R202-791 might arise from the phenylmethyl piperidinyl group in benidipine. This group establishes more interactions with the channel and appears to tolerate substitutions at positions T1056 and Q1060, especially in N- and T-type Ca_V_ channels.

Several dihydropyridine drugs binding L-type calcium channels complex structures have been resolved in previous study^21,22,66–68^, including amlodipine bound Ca_V_Ab (7KMD), nimodipine bound Ca_V_Ab (7KMF), nifedipine bound Ca_V_1.1 (6JP5), amlodipine bound Ca_V_1.1 (7JPX), (S)-Bay K 8644 bound Ca_V_1.1 (6JP8) and (R)-Bay K 8644 bound Ca_V_1.1 (7JPW). When Ca_V_1.2^BEN^ and these structures are superimposed, a same binding pocket in DIII-DIV fenestration are revealed to accommodate benidipine in Ca_V_1.2 and amlodipine/(S)-Bay K 8644/(R)-Bay K 8644 in Ca_V_1.1 (Supplementary Fig. 8a–c). However, benidipine binding site do not overlap with binding sites of amlodipine and nimodipine in Ca_V_Ab (Supplementary Fig. 8d–f), and the residues involved in interaction are not conserved as well.

L-type selective dihydropyridine (DHP) inhibitors and L/T-selective DHP inhibitors are cataloged in Supplementary Table 2. The structural similarities among Class 1, 2, and 3 necessitate collective consideration. Upon scrutinizing their chemical structures and inhibitory selectivity, we identified primary distinctions in functional group modifications, specifically additional alterations on the C3 and C4 benzene rings. Within Class 1 and Class 2, with the exception of three instances (cilnidipine, amlodipine, nimodipine), all DHP derivatives predominantly featured smaller, relatively hydrophilic C3 groups for L-type selective compounds, while L/T-type selective compounds displayed larger and hydrophobic C3 groups. For Class 4 and Class 5 compounds, characterized by a modification into a ring at the C5/C6 position, the sole difference between the L-type selective Class 4-1 and L/T-type selective Class 4-4 compounds lies in the presence of a benzoic acid group on the C4 benzene ring in Class 4-1, while Class 4-4 has a hydroxyl group at the corresponding position. Thus, for compounds with C5-C6 rings, the hydrophobic nature of C3 modification does not exert a significant impact; the pivotal factor influencing differences is the presence of an additional aromatic group modification on the C4 benzene ring.

To further investigate interactions among compounds of varied selectivity on L-type and T-type channels, CaV1.2^BEN^ served as a docking template. L-type selective compounds from Class 1 were docked onto L-type channel Ca_V_1.2, while L/T-type selective compounds from Class 1 were docked onto both L-type channel Ca_V_1.2 and T-type channel Ca_V_3.1. Irrespective of L-type or L/T-type selectivity, these compounds bound to the D_III_-D_IV_ fenestration in Ca_V_1.2 or Ca_V_3.1, exhibiting a favorable superposition of the dihydropyridine backbone (Supplementary Fig. 9b, c). Both L-type selective and L/T-type selective DHPs docked to Ca_V_1.2 formed hydrogen bonds with T1056, Q1060, and S1132. However, the C3 group of L-type selective DHPs docked to Ca_V_1.2 is confined near M1509 within the hydrophobic pocket formed by D_III_S6 and D_IV_S6. In contrast, L/T-type selective DHPs docked to Ca_V_3.1, while unable to form hydrogen bonds at the corresponding positions, not only occupy the binding pocket formed by D_III_S6 and D_IV_S6 but also extend to the hydrophobic pocket formed by D_III_S5 and D_IV_S5, akin to the position occupied by L/T-type selective DHPs docked to Ca_V_1.2. This suggests that the ability of the C3 group to occupy the binding pocket formed by D_III_S5 and D_IV_S5 may be pivotal in determining the L-type and L/T-type selectivity of DHPs. In the case of cilnidipine, its C3 benzenyl group occupies the C5 position in the Ca_V_1.2 binding pocket, possibly due to the planar rigidity of the phenyl group preventing it from fitting into the C3 position. The excessively large C5 group may clash with F1400 in Ca_V_3.1, elucidating why cilnidipine, despite its large and hydrophobic C3 group, cannot inhibit T-type channels. In the case of the other exception, amlodipine docked in Ca_V_3.1, the C2 amino group extends towards the pore and forms potential interactions with hydrophilic residues K1462 and Q1868 near the pore, compensating for the modest interaction between the C3 group and Ca_V_3.1. Given that L/T-type selective DHPs also exhibit different affinities for various T-type channels, such as nimodipine’s preference for inhibiting Ca_V_3.2 and Ca_V_3.3 over Ca_V_3.1 (ref. ^64^), we aligned the Ca_V_3.3 structure with the Ca_V_3.1 structure. The alignment results revealed that the hydrophobic pockets formed by D_III_S5 and D_IV_S5 in Ca_V_3.3 are smaller than those formed in Ca_V_3.1, potentially explaining why nimodipine produces distinct inhibitory effects on T-type channels with different α1 subunits.

## Methods

### Electrophysiology

Whole-cell voltage-clamp recordings were carried out using human embryonic kidney 293 T (HEK293T) cells, which were cultured in Dulbecco’s Modified Eagle Medium (DMEM, Gibco) supplemented with 10% (v/v) fetal bovine serum (FBS, PAN-Biotech) at 37 °C supplemented with 5% CO_2_. Three distinct recombinant baculoviruses encoding Ca_V_1.2, α2δ1, and β2 were used to coinfect HEK293T cells once they reached a confluence of 40–60%. After 8−12 h, transfected cells were re-plated on 8 × 8 mm coverslips, and sodium butyrate was added to the culture at a final concentration of 0.5−2.5 mM to improve protein expression. Whole-cell recordings of activation curves were performed from isolated GFP-positive HEK293T cells at 20−28 h after transfection with recombinant baculoviruses. Briefly, the external solution was composed of 105 mM NaCl, 30 mM TEA-Cl, 10 mM BaCl_2_, 10 mM HEPES pH 7.3, 10 mM D-glucose, 1 mM MgCl_2_, 5 mM CsCl, and 318 mOsm/L. The pipette solution contained 135 mM K-gluconate, 10 mM HEPES pH 7.2, 5 mM EGTA, 2 mM MgCl_2_, 5 mM NaCl, 4 mM Mg-ATP, and 295 mOsm/L^69^. Pipettes were pulled to obtain final resistances of 2−6 MΩ with a Sutter P-97 puller and heat-polished before employment. For measuring the inhibitory effect of tetrandrine and benidipine, the series resistance was 2−10 MΩ and was compensated 80–90%; and for measuring the steady-state activation curve, the series resistance was <5 MΩ. The series resistance was not compensated in these experiments. All recordings were collected with an Axoclamp 700B amplifier and Digidata 1440 A (Molecular Devices). Signals were digitized at 10 kHz and low-pass filtered at 2 kHz. Data were analyzed with Clampfit 10.7.

To measure the inhibition curves, 2 μg endotoxin-free plasmids of wild-type (WT) or mutants generated by PCR expressing Ca_V_1.2, α2δ1, and β2 were transiently transfected using 1.2 μg Lipofectamine 2000 Reagent (Thermo Fisher Scientific). For curves determining, re-plated cells were held at −100 mV, and then depolarized at +10 mV to elicit inward currents. To ensure stable currents during measurement, a series of traces were pre-recorded at a specific time interval without drug application for each construct. The duration of stable currents for WT, T1056Y, Q1060M, and Q1060F was determined as ~10 min, meanwhile the duration of stable currents for N741W and N1179W was determined as ~3 min. Consequently, the depolarization time interval is set every 1 min for benidipine and every 20 s for tetrandrine. Perfusions applied using a gravity-driven system. The inhibition curve of benidipine was determined by first perfusing with the external solution and then with various concentrations of benidipine until the current amplitude reached a steady-state level. The inhibition curve of tetrandrine was determined by applying serial dilutions of tetrandrine at different concentrations for 20 s each. Data analyses were performed using Origin 2022 (Origin Lab Corporation), GraphPad Prism 9 (GraphPad Software, Inc.) and Adobe illustrator 2018. Inhibition curves were generated using a Hill equation.

1 $$\frac{I}{{I}_{\max }}=\frac{1}{1+{(\frac{C}{{{IC}}_{50}})}^{H}}$$

where *I* is the current at different drug concentrations, *I*_max_ is the maximal current of Ca_V_1.2 without drug applied, [*C*] is the concentration of drugs, IC_50_ is the half-maximal inhibitory concentration and *H* is the Hill coefficient.

### Clone, expression and purification of human Ca_V_1.2-α2δ1-β2b complex

DNA fragments encoding Ca_V_1.2 α1C, α2δ1, and β2b were amplified from a human cDNA library and subcloned into the pEG BacMam vector for co-expression in mammalian cells. For Ca_V_1.2, the C-terminus was tandemly fused with a superfolder GFP (sfGFP) and a Twin-Strep affinity tag. The fluorescent protein mCherry and Twin-Strep tag were fused to the N-terminus of wild type α2δ1 and β2b subunits, respectively. Primers used for cloning and introducing mutations are provided in Supplementary Table 3. The Bac-to-Bac system (Invitrogen, USA) was used to produce recombinant baculovirus in *sf9* cells (Thermofisher, 10902096). The HEK293F cells (Thermofisher, 11625019) at density of ~2 × 10^6^ cells per ml were infected with 2% (v/v) P2 recombinant baculovirus and subsequently cultured at 37 °C with 5% CO_2_. After 12 h, 10 mM sodium butyrate was added to the medium to enhance protein expression. The cells were harvested 60 h after infection.

The cell pellets were resuspended and grinded on ice in a Dounce homogenizer using buffer D containing 20 mM HEPES pH 7.5, 150 mM NaCl, 1 mM CaCl_2_, 5 mM β-mercaptoethanol (β-ME), 2 μg/mL aprotinin, 1.4 μg/mL leupeptin, and 0.5 μg/mL pepstatin A. The membrane was enriched by centrifugation at 110,000 g for 40 min at 4 °C. The collected membrane pellets were solubilized in buffer D supplemented with 1% (w/v) n-dodecyl-β-D-maltoside (DDM), 0.15% (w/v) cholesteryl hemisuccinate (CHS) (Anatrace, USA), 2 mM adenosine triphosphate (ATP), and 5 mM MgCl_2_ for 2 h at 4 °C. The solubilized membrane was subjected to centrifugation at 110,000 g for 40 min at 4 °C, and the supernatant was passed through a 0.22 μm filter before being applied to a streptavidin agarose column for protein affinity purification. Following loading of the sample, the column was washed with buffer D containing additional 0.025% DDM to remove nonspecific bound proteins. The Ca_V_1.2 -α2δ1-β2b complex was then eluted with a buffer containing 5 mM desthiobiotin, 20 mM HEPES pH 7.5, 150 mM NaCl, 1 mM CaCl_2_, 5 mM β-ME, and 0.007% glyco-diosgenin (GDN) (Anatrace, USA). The elution was concentrated to a final volume of 1 mL with 100-kDa Millipore Tube (Merck Millipore, Germany) and further purified through size exclusion chromatography (SEC) using a Superose 6 Increase 10/300 GL column (GE Healthcare, USA) equilibrated with a buffer containing 20 mM HEPES pH 7.5, 150 mM NaCl, 1 mM CaCl_2_, 5 mM β-ME, and 0.007% GDN. The peak fractions at ~12.5 mL were collected and concentrated to 7 mg/mL for cryo-EM grids preparation. For the tetrandrine/benidipine bound Ca_V_1.2 complex, 10 μM concentration of tetrandrine (MedChemExpress (Monmouth Junction, NJ, USA)) was supplied throughout the expression and purification of Ca_V_1.2 complex and 1 μM concentration of benidipine (MedChemExpress (Monmouth Junction, NJ, USA)) was supplied in the membrane solubilization process. A final concentration of 100 μM tetrandrine/benidipine was added to the cryo-EM sample and incubated for 40 min on ice before application in grids.

### Cryo-EM sample preparation and data collection

Quantifoil 1.2/1.3 Cu 300 mesh grids were glow-discharged for 60 s under H_2_-O_2_ condition with a Solarus plasma cleaner (Gatan, USA) before use. The grids were applied with a 2.5-μL droplet of protein sample at 4 °C and 100% humidity, and then snap-frozen in liquid ethane cooled by liquid nitrogen using a Vitrobot Mark IV (Thermo Fisher Scientific, USA). The grids sample was stored in liquid nitrogen before being checked on the electron microscopy instrument.

Cryo-EM data were collected on a 300-kV Titan Krios (Thermo Fisher Scientific, UAS) equipped with a K2 Summit direct electron detector (Gatan, USA) and a GIF-Quantum LS energy filter. The energy filter slit width was set to 20 eV. Movie stacks were acquired using SerialEM^70^ at a calibrated magnification of 130,000× in the super-resolution mode, with defocus values ranging from −1.2 to −2.2 μm. The pixel size on motion-corrected micrographs was 1.04 Å. Each movie stack was dose-fractioned in 32 frames, yielding a total accumulated dose of 60 e^−^/Å^2^. The dose rate was set to 9.6 e^–^/pixel/s.

### Cryo-EM data processing

For the data processing of Ca_V_1.2^apo^, a total of 1278 movie stacks were motion-corrected and dose-weighted using MotionCorr2 with 5 × 5 patches^71^. Contrast transfer function (CTF) estimation was performed using Gctf^72^. Particles that picked using Blob picker, Template picker, and Topaz picker in cryoSPARC^73^ were combined, and duplicates were removed, yielding a total of 524k particles. Then, the particles were extracted into Relion 3.1 for guided multi-reference 3D classification against one good map which was generated by low pass filtering the high-resolution map of Ca_V_2.2 (EMDB-31958) to 8 Å and 4 biased maps^74^. Particles from the class 1, which accounts for 47.1% of total particles, were selected and subjected to a single reference 3D classification to further remove poor particles, giving rise to one class with discernible structural features of α1, α2δ1, and β subunits as well as CTD. Particles belonging to this class were submitted for following 3D auto refinement, Bayesian Polish, and CTF refinement, yielding a 3.9-Å resolution map with clearly resolved transmembrane helices. To improve the quality of the map, a protein-only mask was used to avoid over-fitting of the detergent micelles in the subsequent 3D classification without particle alignment. The best class containing 35k particles was then imported back into cryoSPARC and subjected to Non-uniform Refinement, generating a final map reported at 3.5-Å resolution according to golden standard Fourier shell correlation (GSFSC) criterion.

A similar strategy was applied in the data processing of Ca_V_1.2^TET^ and Ca_V_1.2^BEN^. Specifically, a total of 449k and 337k particles were picked from 979 and 444 micrographs, respectively. The final maps of Ca_V_1.2^TET^ and Ca_V_1.2^BEN^ were reported at 3.4 Å and 3.3 Å, respectively. A diagram of data processing is summarized in Supplementary Fig. 2.

### Model building

To build the atomic model of Ca_V_1.2^apo^, we extracted rabbit α1, α2δ1, and human β3 subunits from the structures of the rabbit Ca_V_1.1 complex (5GJV)^33^ and Ca_V_2.2 complex (7MIJ)^75^ and generated homology models of the α1, α2δ1, and β2b subunits of Ca_V_1.2 using phenix.sculptor program based on sequence alignment. The resulting models of three subunits were fitted into the map of the Ca_V_1.2^apo^ complex as rigid bodies using the UCSF Chimera. Then, the model was manually adjusted in COOT^76^ iteratively, including the refinement of the main chain and side chains of residues. Phospholipid molecules were manually placed in the strip-shaped densities in both leaflets of the lipid bilayers and fenestrations of the pore domain of the Ca_V_1.2 channel. Structure refinement was performed using phenix.real_space_refine application in PHENIX^77^ in real space with secondary structure and Ramachandran restraints.

For the model building of Ca_V_1.2^TET^ and Ca_V_1.2^BEN^, the Ca_V_1.2^apo^ structure was used as the starting model and was fitted into the EM maps as a rigid body. The two-dimensional (2D) structures of tetrandrine and benidipine were downloaded from PubChem in SDF format, followed by the generation of 3D models and refinement restraints in phenix.ligand_eLBOW. The drug molecules were docked into the EM map and refined according to the corresponding density. All the manually adjusted models were then subjected to real-space refinement using PHENIX.real_space_refine.

All figures were prepared with software PyMOLl^78^ or UCSF Chimera^79^.

### Docking

To investigate the interaction modes of various dihydropyridines (DHPs) in T-type and L-type calcium channels, a docking study was conducted utilizing the AutoDock Tools package (version 1.5.6)^80^ and AutoDock Vina (version 1.1.2)^81^. In the docking simulation, L-type selective inhibitors (Nifedipine, Nitrendipine, Felodipine, Cilnidipine) and non-selective inhibitors (Benidipine, Nimodipine, Manidipine, Amlodipine)^64,82^ were employed as ligands, while Ca_V_1.2, Ca_V_3.1 (6KZP)^83^, and Ca_V_3.3 (7WLI)^52^ were utilized as receptors. The receptors and ligand were independently optimized and prepared as pdbqt format files required for docking. Given that the DHP binding pocket in the apo-state structure comprises only lipid and the site is considerably narrower compared to Ca_V_1.2, the lipid and ligand was removed and the side chain dihedral angles of DHP binding pocket were adjusted. The docking grids for Ca_V_1.2 were generated using enclosing boxes centered on benidipine, whereas the docking grids for Ca_V_3.1 (or Ca_V_3.3) were created using enclosing boxes centered on the residue L872 (or L769) involved in the DHP binding pocket. The processed ligands were subsequently docked into the two receptors. Only docking results with ligand conformations in a ‘claw’ shape configuration and biased towards DIII which analogous to the Ca_V_1.2-benidipine complex that we have resolved were retained.

### Reporting summary

Further information on research design is available in the Nature Portfolio Reporting Summary linked to this article.

### Supplementary information

Supplementary Information

Peer Review File

Description of Additional Supplementary Files

Supplementary Data 1

Reporting Summary

### Source data

Source Data

## Data Availability

The data that support this study are available from the corresponding authors upon request. The three-dimensional cryo-EM density maps of the Ca_V_1.2 complex in the apo state, tetrandrine bound state, and benidipine bound state have been deposited in the Electron Microscopy Data Bank under the accession codes EMD-34880, EMD-34891, and EMD-34892, respectively. The coordinates for the corresponding complexes have been deposited in Protein Data Bank under accession codes 8HLP (Ca_V_1.2^apo^), 8HMA (Ca_V_1.2^TET^), and 8HMB (Ca_V_1.2^BEN^). The model used for starting Ca_V_1.2 complex model-building and docking is available in the PDB under the PDB ID 5GJV (rabit Ca_V_1.1), 7MIJ (human Ca_V_2.2), 6KZP (human Ca_V_3.1 bound Z944), and 7WLI (human Ca_V_3.3). Sequence of human Ca_V_1.2 α1C, α2δ1 and β2b are available in Universal Protein Resource (Uniprot) databases under accession codes Q13936-1 [https://www.uniprot.org/uniprotkb/Q13936/entry] (CACNA1C), P54289-1 [https://www.uniprot.org/uniprotkb/P54289/entry] (CACNA2D1), and Q08289-3 [https://www.uniprot.org/uniprotkb/Q08289/entry] (CACNB2). The source data underlying Fig. 3f, Supplementary Figs. 1a, c, and 7c are provided as a Source Data file. Source data are provided with this paper.
