# Supplementary material for: Structural bases of inhibitory mechanism of CaV1.2 channel inhibitors
Source: Nat Commun. 2024 Mar 30;15:2772. doi: 10.1038/s41467-024-47116-8 (PMC10981686; doi:10.1038/s41467-024-47116-8)
Supplement: Supplementary file 1 — Supplementary Information [file 41467_2024_47116_MOESM1_ESM.pdf]

Supplementary Information for

Structural Bases of Inhibitory Mechanism of $Ca_V1.2$ Channel Inhibitors

Yiqing Wei, Zhuoya Yu, Lili Wang, Xiaojing Li, Na Li, Qinru Bai, Yuhang Wang, Renjie Li, Yufei Meng, Hao Xu, Xianping Wang, Yanli Dong, Zhuo Huang, Xuejun Cai Zhang, Yan Zhao

This file contains Supplementary Figure 1-9, Supplementary Table 1-3 and Supplementary Reference.

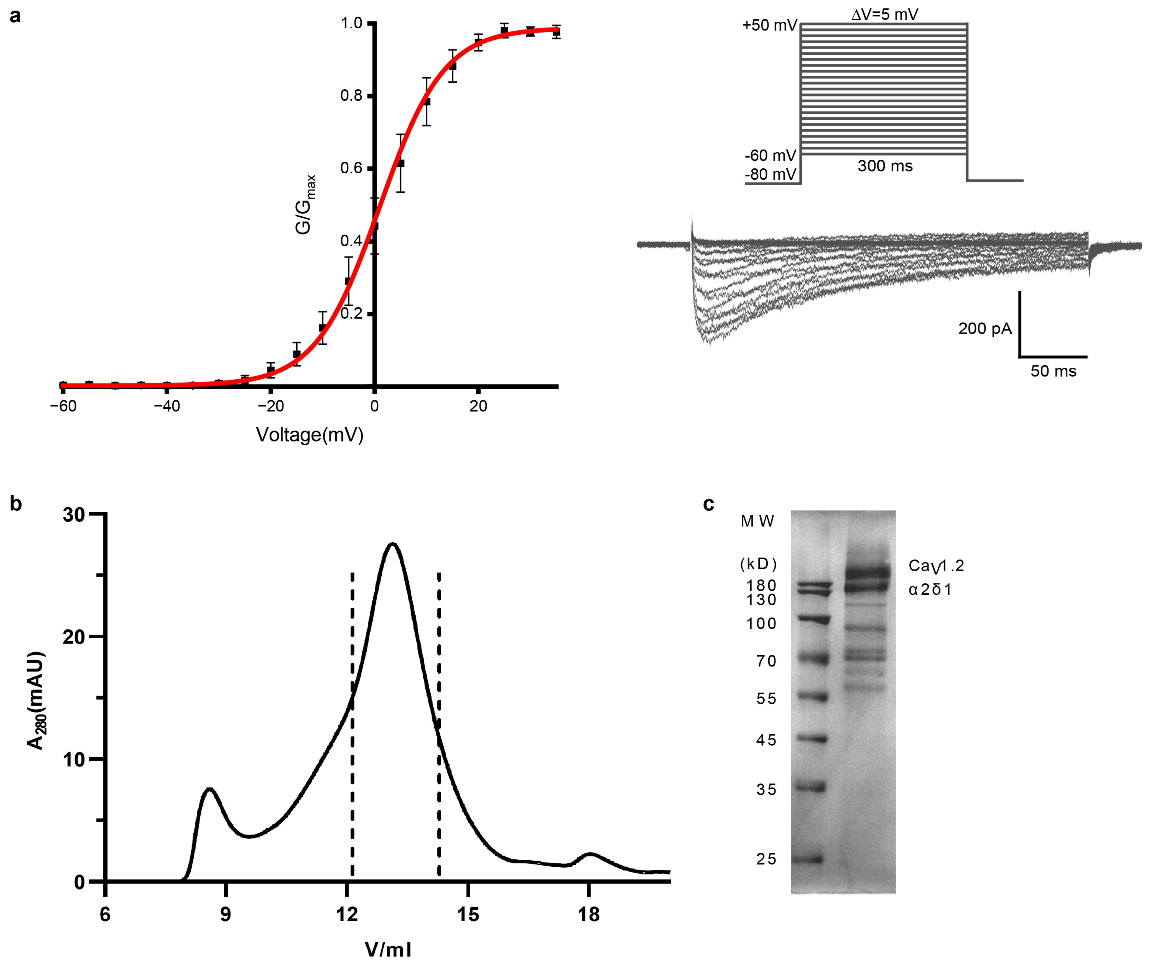

Supplementary Fig. 1 Functional characterization and purification of the $Ca_V1.2$.

a. Normalized conductance-voltage (G/V) relationship for the $Ca_V1.2$. Whole cell currents of HEK293T cells transfected with $Ca_V1.2$ were recorded with 300-ms depolarizing pulses between -60 and 50 mV in steps of 5 mV from a holding potential of -80 mV. The data were fitted with the Boltzmann equation, yielding half-activation voltage ($V_{1/2}$) of 1.0±0.5 mV (n=3 biologically independent experiments). **b.** Representative elution profile of the purified $Ca_V1.2$ proteins by size-exclusion chromatogram (Superose 6 increase). Peak fractions (marked within black dashed lines) are pooled and concentrated for cryo-EM study. **c**. Coomassie-blue-stained SDS-PAGE gel of the purified $Ca_V1.2$ complex. Components of the complex are labeled. The experiments were repeated independently for more than 3 times with similar results. Source data are provided as a Source Data file.

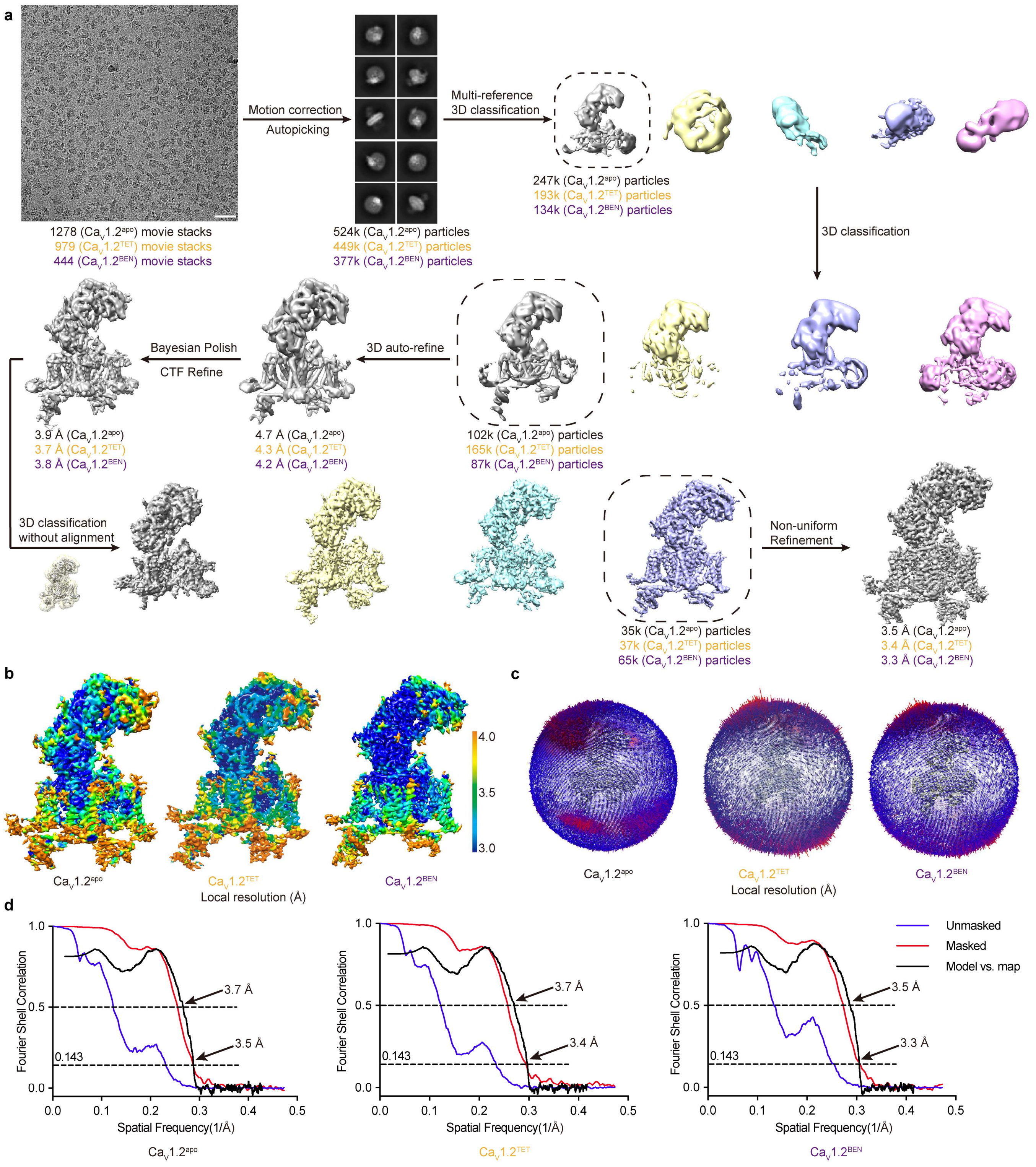

Supplementary Fig. 2 Cryo-EM data processing of Ca$_V$1.2^{apo}, Ca$_V$1.2^{TET}, and Ca$_V$1.2^{BEN}.

a. Flowchart of cryo-EM data processing. Representative raw cryo-EM micrograph and 2D class averages shown distinct secondary structure features from different views of Ca$_V$1.2^{apo}, respectively (Bar = 400 Å). Several rounds of 3D classifications are conducted to clean particles, followed by Bayesian Polish and CTF Refine to improve image quality. The resolution and number of particles during data processing are labeled (Ca$_V$1.2^{apo} in black, Ca$_V$1.2^{TET} in orange,

and $Ca_V1.2^{BEN}$ in purple) and details can be found in Materials and Methods.The final map was reported at 3.5-Å for $Ca_V1.2^{apo}$, 3.4-Å for $Ca_V1.2^{TET}$, and 3.3-Å for $Ca_V1.2^{BEN}$ according to the GSFSC criterion, respectively. **b.** Electron density map colored by local resolution values. From left to right: $Ca_V1.2^{apo}$, $Ca_V1.2^{TET}$, and $Ca_V1.2^{BEN}$. The colour scale from blue to orange indicates the resolution from 3.0-Å to 4.0-Å. **c.** Angular distribution histogram of the map of $Ca_V1.2^{apo}$ (left), $Ca_V1.2^{TET}$ (middle), and $Ca_V1.2^{BEN}$ (right). **d.** Fourier Shell Correlations (FSC) of the final map of $Ca_V1.2^{apo}$ (left), $Ca_V1.2^{TET}$ (middle), and $Ca_V1.2^{BEN}$ (right), calculated between two independently refined half-maps before (blue) and after (red) post-processing. The FSC curve calculated between the cryo-EM density map and the structural model are shown in black.

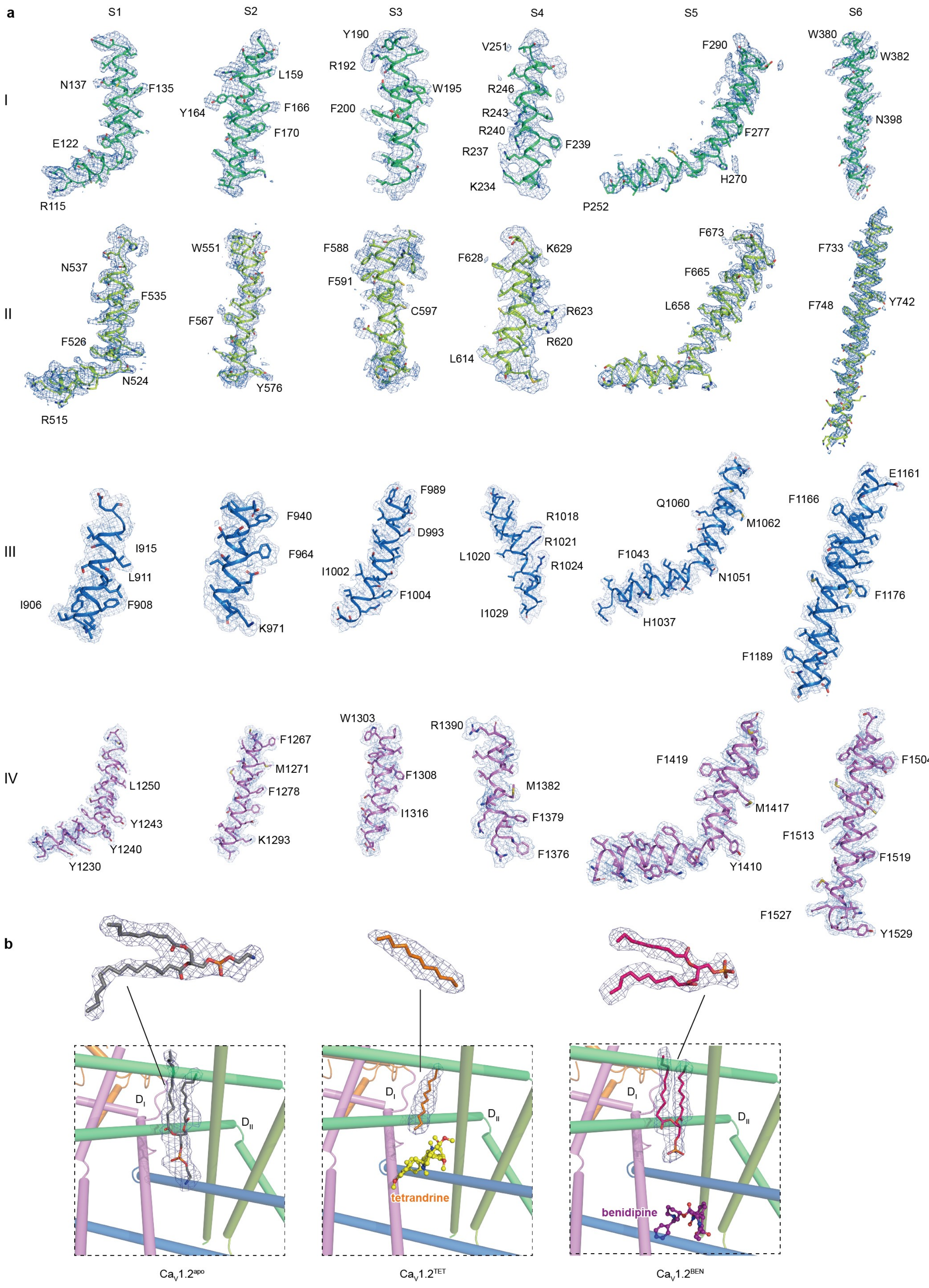

Supplementary Fig. 3 Cryo-EM map of the Ca$_V$1.2 structure.

a. The cryo-EM density map and atomic model of S1-S6 segments in the four repeats of Ca$_V$1.2^{apo}. The side chains of key residues are labeled. The cryo-EM maps are shown as blue mesh. **b.** The cryo-EM density maps of the lipid molecules entering the fenestration site

between DI and DII in $Ca_V1.2^{apo}$ (left), $Ca_V1.2^{TET}$ (center), and $Ca_V1.2^{BEN}$ (right). The domains of $Ca_V1.2$ are colored as D_I in deep green, D_{II} in light green, D_{III} in deep blue, and D_{IV} in mauve.

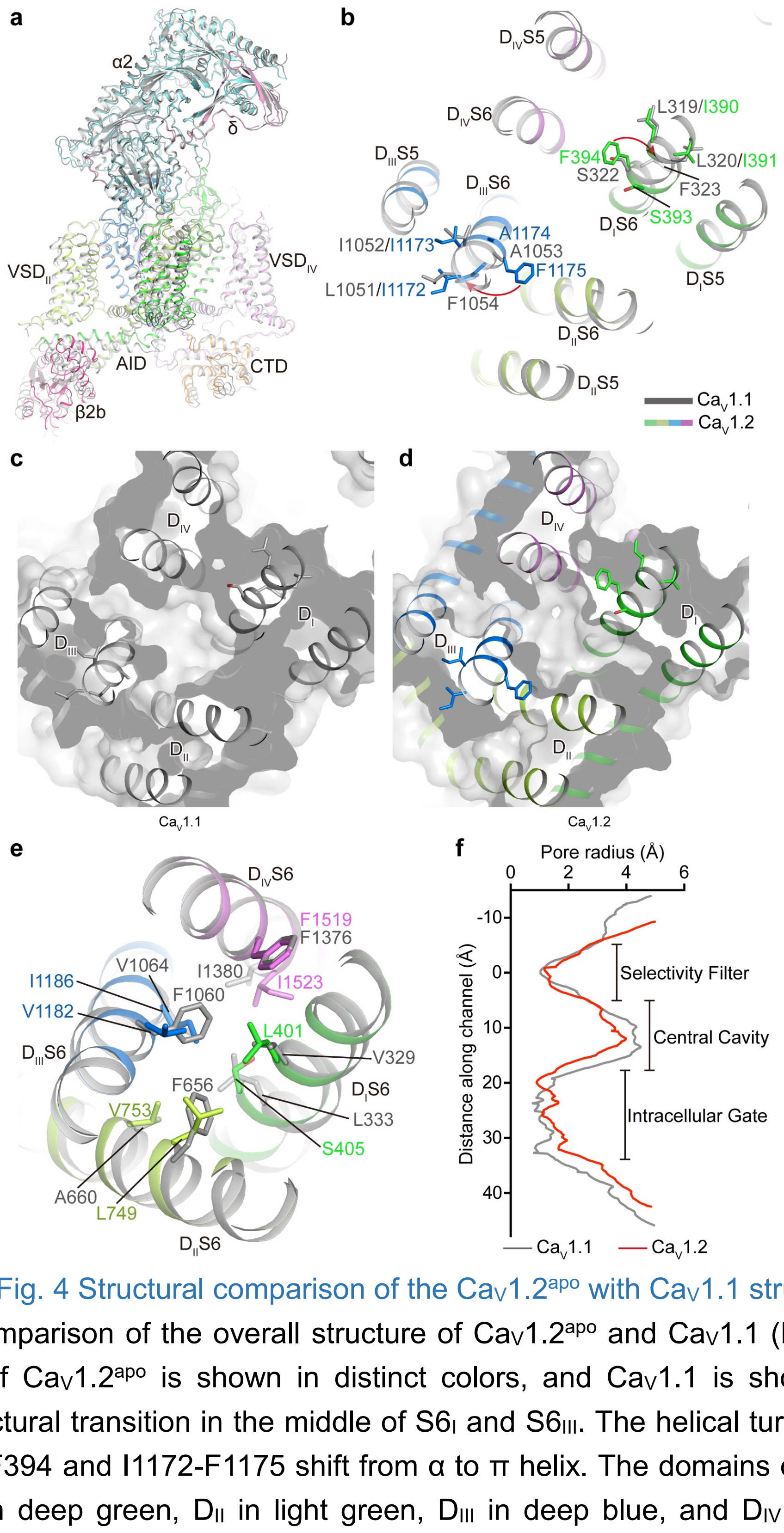

Supplementary Fig. 4 Structural comparison of the $Ca_V1.2^{apo}$ with $Ca_V1.1$ structure.

a. Structural comparison of the overall structure of $Ca_V1.2^{apo}$ and $Ca_V1.1$ (PDB ID:5GJW). The structure of $Ca_V1.2^{apo}$ is shown in distinct colors, and $Ca_V1.1$ is shown in gray. **b.** Secondary structural transition in the middle of $S6_I$ and $S6_{III}$. The helical turns consisting of residues L390-F394 and I1172-F1175 shift from α to π helix. The domains of $Ca_V1.2^{apo}$ are colored as D_I in deep green, D_{II} in light green, D_{III} in deep blue, and D_{IV} in mauve. **c**.**d.** Extracellular view of gate composed of S6 in $Ca_V1.1$ (**c**) and $Ca_V1.2$ (**d**). **e**. The intracellular gate formed by four S6 helices viewed from the extracellular side. The structure of $Ca_V1.2^{apo}$ is shown in distinct colors, and $Ca_V1.1$ is shown in gray. **f**. Plot of pore radii for the $Ca_V1.1$(PDB ID:5GJW) and $Ca_V1.2^{apo}$ complex.

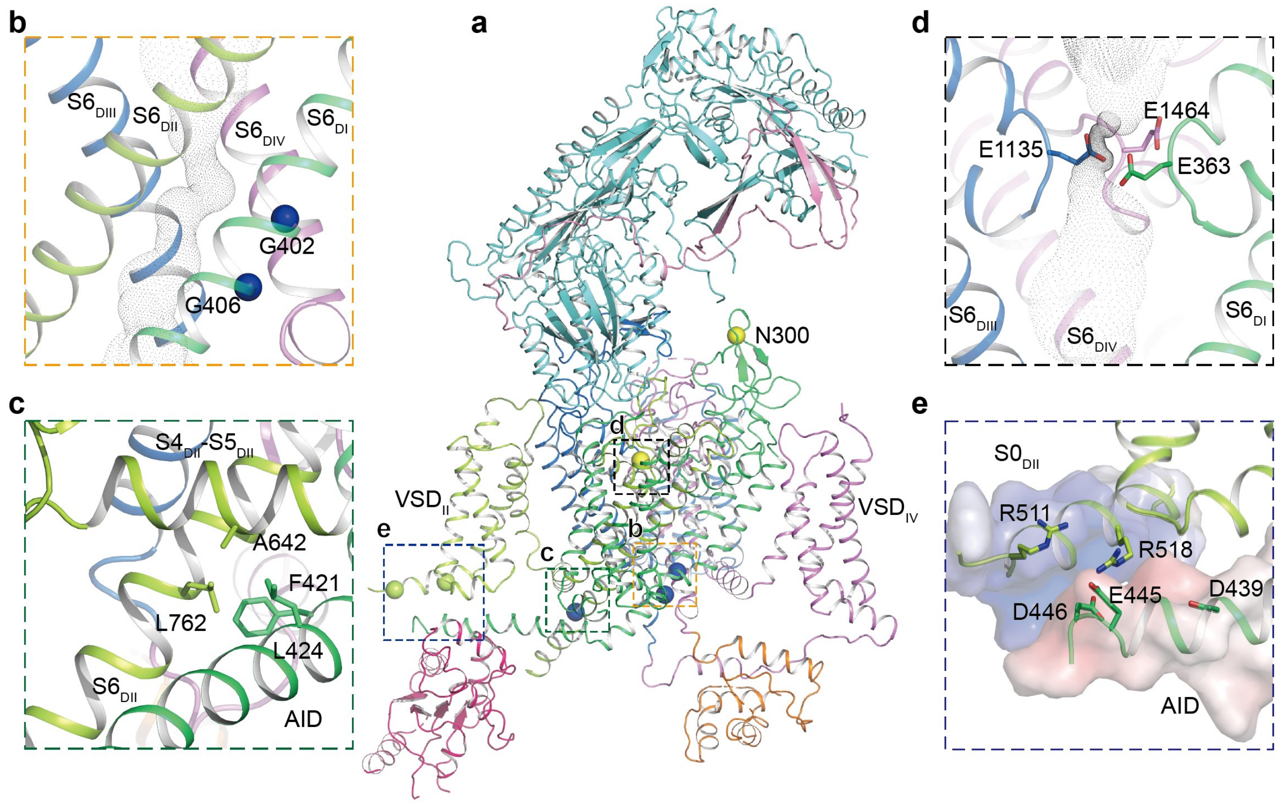

Supplementary Fig. 5 Details of gain of function and loss of function mutations.

a. The distribution of pathogenic mutations of N300, G402/G406, R511/R518, L762 and E1135 on the $Ca_V1.2^{apo}$ structure. N300 is located at extracellular loop adjacent to α2δ1 subunit. The α1 subunit is colored in deep green (D_I), light green (D_{II}), deep blue (D_{III}), mauve (D_{IV}), and orange (C-terminus), respectively. The β2b, α2, and δ1 subunits are colored in magenta, turquoise, and pink, respectively. **b.** The G402 and G406 are located at same face of the S6 helix. **c.** The L762 is located at $S6_{DII}$, facing $S4_{DII}$-$S5_{DII}$. **d.** The E1135 is positioned on the selectivity filter. **e.** The R511 and R518 are involved in electrostatic interactions with D439, D445 and D446 on the AID.

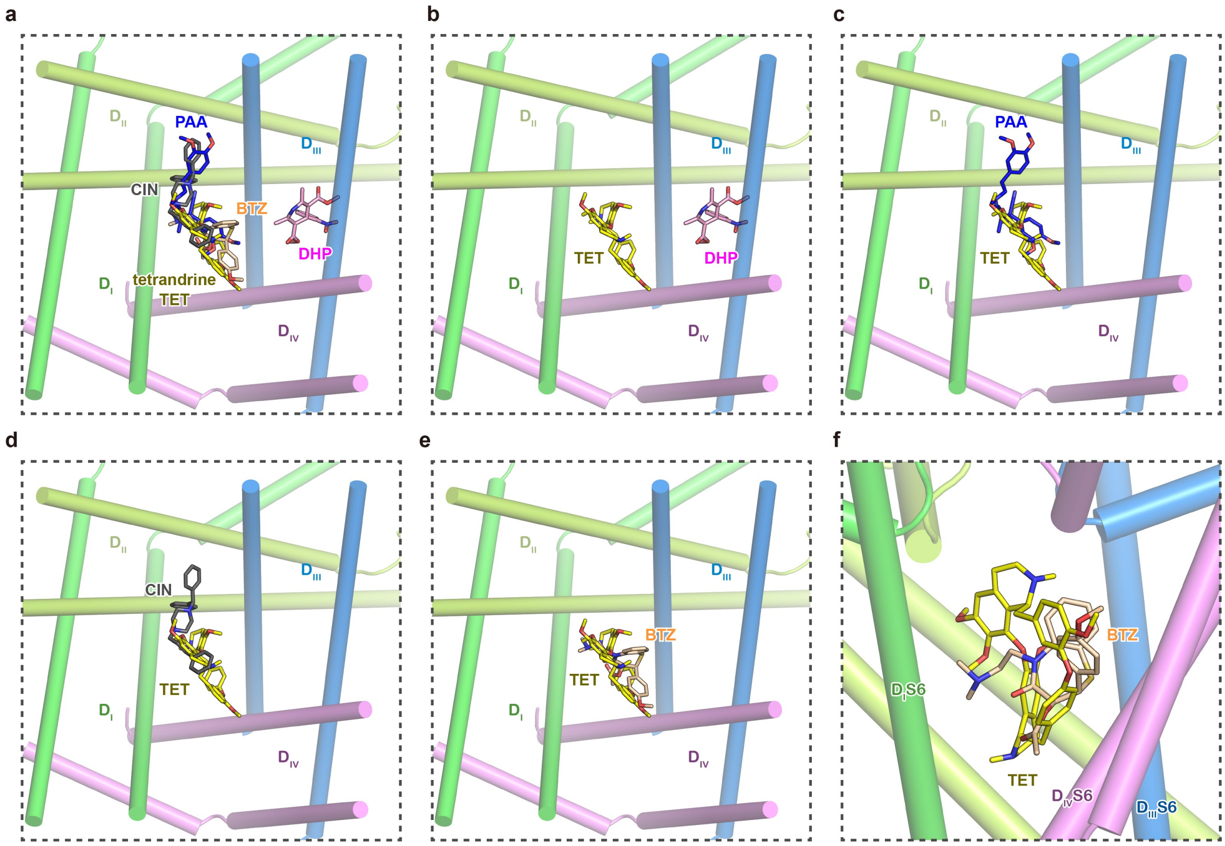

Supplementary Fig. 6 Binding mode of pore blocker tetrandrine compared with other antagonists.

a. The binding mode of tetrandrine (TET) compared with other antagonists. $Ca_V1.2^{TET}$ is shown as scaffold for comparison, tetrandrine is colored yellow. The domains of $Ca_V1.2^{TET}$ are colored as D_I in deep green, D_{II} in light green, D_{III} in deep blue, and D_{IV} in mauve. The $Ca_V1.1$ structure bound dihydropyridine class nifedipine (DHP, colored in pink) (PDB ID: 6JP5), phenylalkylamine class verapamil (PAA, colored in blue) (PDB ID: 6JPA), benzothiazine class diltiazem (BTZ, colored in wheat) (PDB ID: 6JPB), and $Ca_V1.3$ structure bound cinnarizine (CIN, colored in grey40) (PDB ID: 7UHF) were aligned to $Ca_V1.2^{TET}$. **b-e.** The binding site of tetrandrine compared with nidedipine, verapamil, cinnarizine, and diltiazem. **f.** Tetrandrine occupies the binding site of diltiazem.

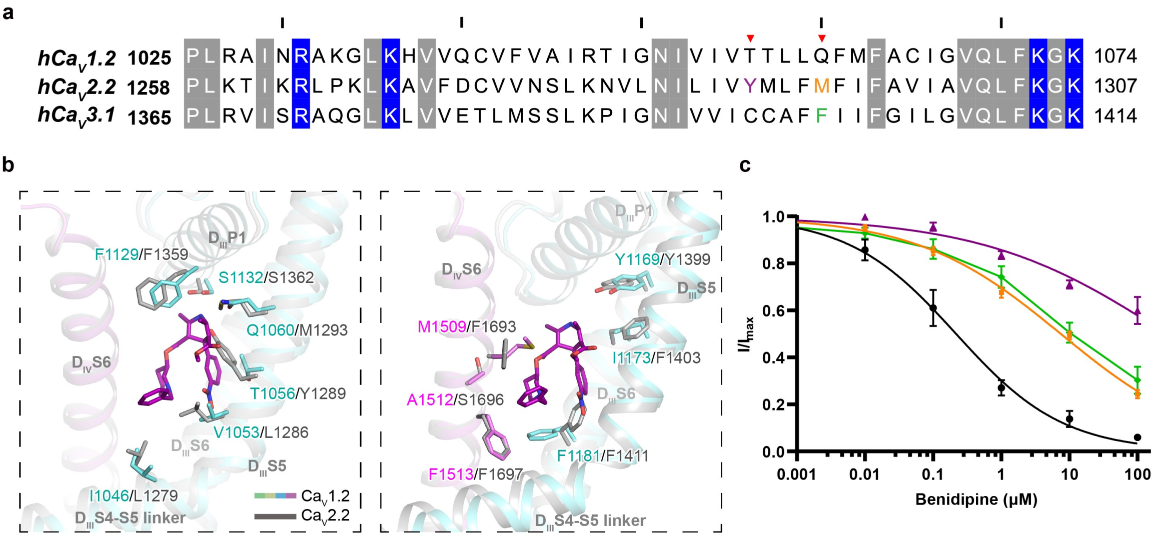

Supplementary Fig. 7 Reduced inhibition of benidipine on $Ca_V1.2$ mutants.

a. Sequence alignment of benidipine binding sites on $Ca_V1.2$, $Ca_V2.2$ and $Ca_V3.1$. Residues contributed to forming hydrogen bonds with benidipine are indicated by red triangles. Conserved alkaline and uncharged residues are shaded in blue and grey, respectively. **b.** Comparison of the benidipine binding sites of $Ca_V1.2$ with $Ca_V2.2$ (PDB ID: 7VFS, colored in gray). **c.** Dose-response curves of benidipine on $Ca_V1.2$, $Ca_V1.2^{T1056Y}$, $Ca_V1.2^{Q1060M}$ and $Ca_V1.2^{Q1060F}$. The IC50 of $Ca_V1.2^{WT}$, $Ca_V1.2^{T1056Y}$, $Ca_V1.2^{Q1060M}$, and $Ca_V1.2^{Q1060F}$ are 216.9 nM, 267.5 μM, 7.5 μM, and 11.7 μM, respectively. Data in curves are and represented as mean ± SEM (n = 4 biologically independent experiments). Source data are provided as a Source Data file.

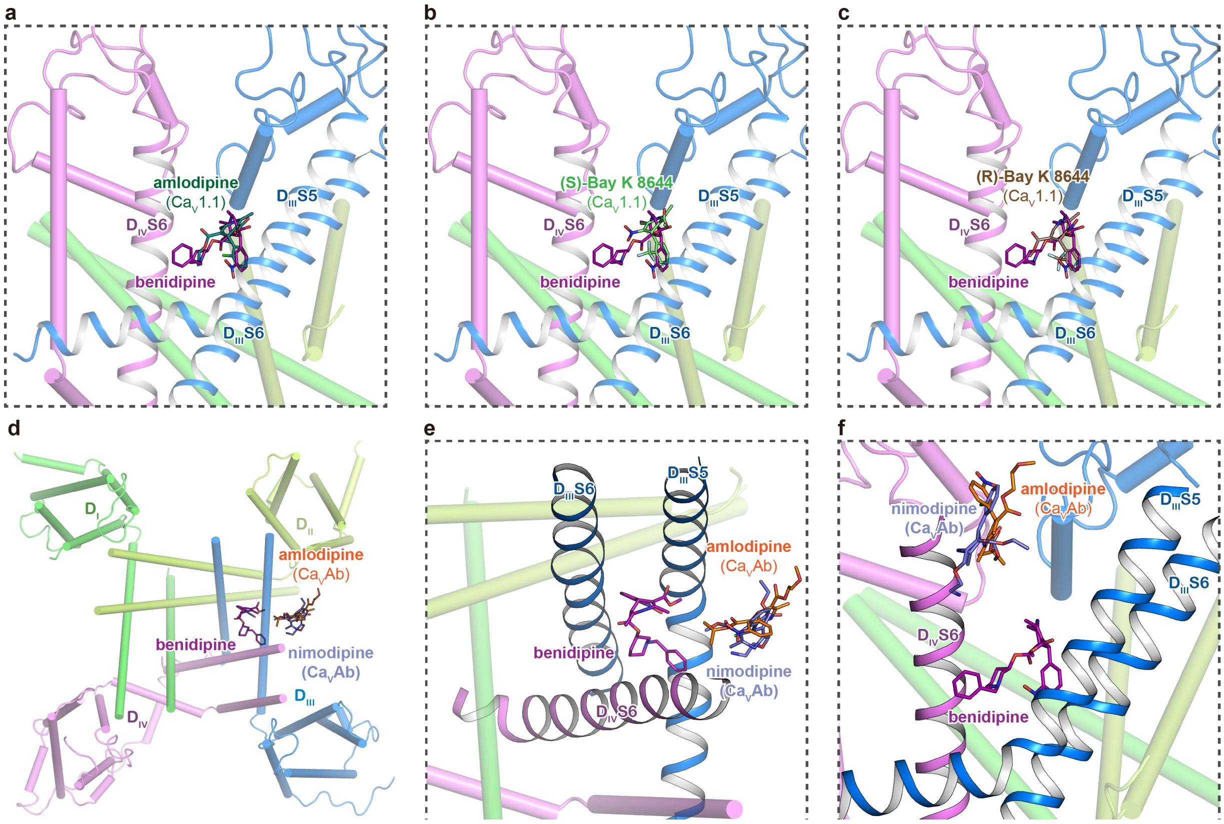

Supplementary Fig. 8 Comparison of benidipine and DHPs resolved in Ca_VAb and $Ca_V1.1$.

a-c. The binding site of benidipine compared with amlodipine / (S)-Bay K 8644 / (R)-Bay K 8644 in $Ca_V1.1$ (PDB ID: 7JPX / 6JP8 / 7JPW). **d.** The binding sites of benidipine compared with other DHPs resolved in Ca_VAb. $Ca_V1.2^{BEN}$ is shown as scaffold for comparison, benidipine is colored in purple. The domains of $Ca_V1.2^{BEN}$ are colored as D_I in deep green, D_{II} in light green, D_{III} in deep blue, and D_{IV} in mauve. The Ca_VAb structure bound nimodipine (PDB ID: 5KMF) and amlodipine (PDB ID: 5KMD) were aligned to $Ca_V1.2^{BEN}$. **e-f**. The binding site of benidipine compared with nimodipine and amlodipine in Ca_VAb from vertical and side view.

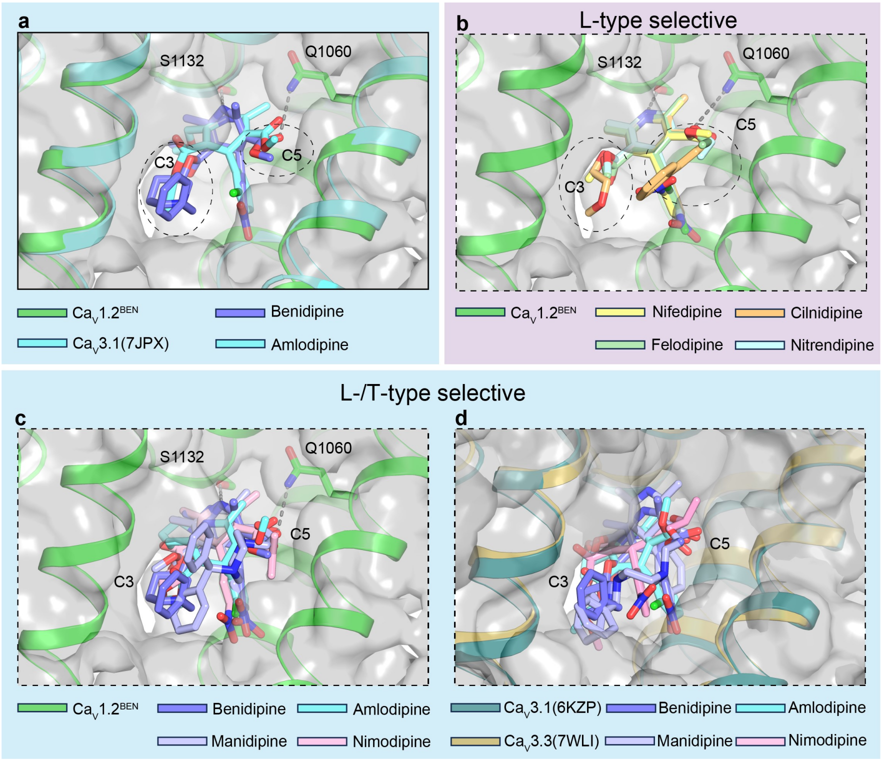

Supplementary Fig. 9 Comparison of the Interactions between Different Dihydropyridine (DHP) and Cav Channels.

a. Display of L/T-type selective inhibitors benidipine in complex with Ca$_V$1.2 and amlodipine in complex with Ca$_V$1.1 (7JPX). **b.** Docking results of L-type selective DHP blocker in Ca$_V$1.2 are displayed. **c.** Display of docking results for L/T-type selective DHP blockers with Ca$_V$1.2. **d.** Display of docking results for L/T-selective blockers with Ca$_V$3.1 (6KZP) and Ca$_V$3.3(7WLI).

Supplementary Table 1. Cryo-EM data collection, refinement and validation statistics

	$Ca_V1.2^{apo}$ (EMDB-34880) (PDB 8HLP)	$Ca_V1.2^{TET}$ (EMDB-34891) (PDB 8HMA)	$Ca_V1.2^{BEN}$ (EMDB-34892) (PDB 8HMB)
Data collection and processing			
Magnification	130,000 ×	130,000 ×	130,000 ×
Voltage (kV)	300	300	300
Electron exposure ($e^{-}/Å^2$)	60	60	60
Defocus range (μm)	−1.2 ~ −2.2	−1.2 ~ −2.2	−1.2 ~ −2.2
Pixel size (Å)	1.04	1.04	1.04
Symmetry imposed	C1	C1	C1
Initial particle images (no.)	524,767	449,890	377,128
Final particle images (no.)	35,628	37,051	65,662
Map resolution (Å)	3.5	3.4	3.3
FSC threshold	0.143	0.143	0.143
Map resolution range (Å)	3.0 ~ 5.0	3.0 ~ 5.0	3.0 ~ 5.0
Refinement			
Initial model used (PDB code)	5GJV/7MIJ	$Ca_V1.2^{apo}$	$Ca_V1.2^{apo}$
Model resolution (Å)	3.5	3.4	3.3
FSC threshold	0.5	0.5	0.5
Map sharpening *B* factor ($Å^2$)	66.2	66.0	65.1
Model composition			
Non-hydrogen atoms	19,067	19,101	19,147
Protein residues	2,332	2,333	2,332
Ligands	18	19	22
B factors ($Å^2$)			
Protein	104.93	104.20	93.62
Ligand	71.42	87.58	59.02
R.m.s. deviations			
Bond lengths (Å)	0.005	0.003	0.008
Bond angles (°)	0.617	0.519	0.832
Validation			
MolProbity score	2.03	1.79	2.12
Clashscore	10.01	7.45	13.23
Poor rotamers (%)	0.00	0.00	0.00
Ramachandran plot			
Favored (%)	91.40	94.42	91.93
Allowed (%)	8.60	5.58	8.07
Disallowed (%)	0.00	0.00	0.00

Supplementary Table 2. Summary of Selectivity of DHPs for L-type and T-type Channels

Compound	Blocker of L-type channels	Blocker of both T-type and L-type channels	References
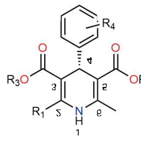 **Class 1**	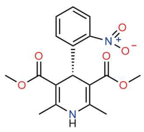 Nifedipine 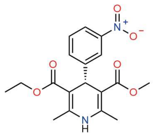 Nitrendipine 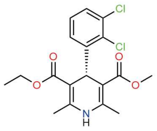 Felodipine 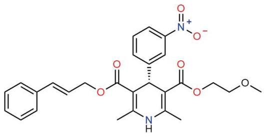 Cilnidipine	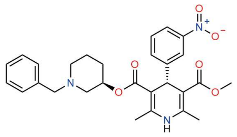 Benidipine 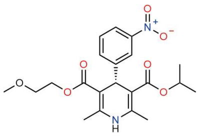 Nimodipine 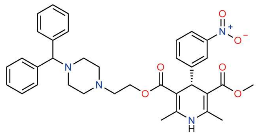 Manidipine 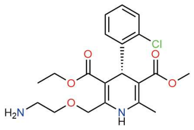 Amlodipine	1, 2
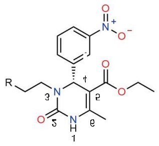 **Class 2**	**1** 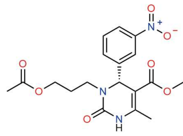	**2** 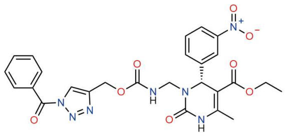 **3** 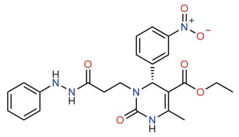	3
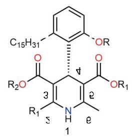 **Class 3**		**1** 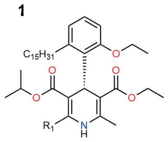 **2** 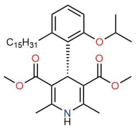	4
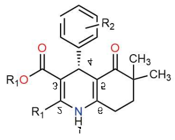 **Class 4**	**1** 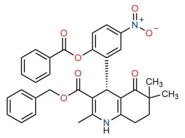 **2** 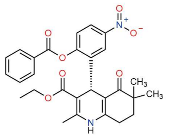	**3** 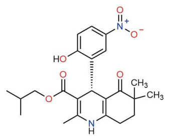 **4** 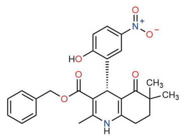 **5** 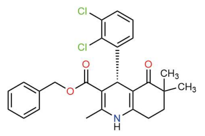 **6** 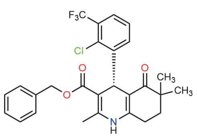	5, 6
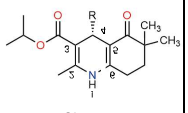 **Class 5**	**1** 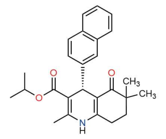	**2** 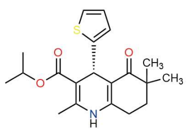 **3** 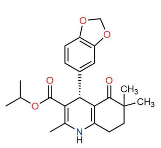	7

On the left is the general structural formula of compound grouped in one class. Bold numbers represent the serial number of compound without official name in same class.

Reference in this table are listed in Supplementary Reference.

Supplementary Table 3. Primers used in this study

Name	Sequence (5' to 3')	Source
Ca_V1.2-pEG-F	TCCCGGTCCGAAGCGCGCGGAATTCatggtcaatgagaatacgaggatgt	RuiBiotech
Ca_V1.2-pEG-R	GGAACAGAACTTCCAGTGCGGCCGCcaggctgctgacgtagaccctgctg	RuiBiotech
Ca_V1.2-splice-exon21/22-F	gtttttaccaccatttttacattagaaattatc	Tsingke
Ca_V1.2-splice-exon21/22-R	ttctaatgtaaaaatggtggtaaaaacaatatc	Tsingke
Ca_V1.2-splice-exon31/32-F	gacgtcattctcagtgaggtaaacccagctgaac	Tsingke
Ca_V1.2-splice-exon31/32-R	ctgggtttacctcactgagaatgacgtcaattatg	Tsingke
β2-pEG-F	TCTGTTCCAGGGGCCCGAATTCatggtccaaagggacatgtc	RuiBiotech
β2-pEG-R	CTGCAGGCTCTAGATTCGAAAtcattggcggatgtaaacatc	RuiBiotech
β2b-pEG-Nt-F	tagctcctcaaactaaatacattattcctgggggttcggcagactcctacactagccgt	Tsingke
β2b-pEG-Nt-R	ttagtttgaggagctataaggcgtctgtcaagcatGAATTCGGGCCCCTGGAACAGAAC	Tsingke
a2d1-pEG-F	AGACTGCAGGCTCTAGATTCGAAAtcataacaggcggtgtgtgctgcca	RuiBiotech
a2d1-pEG-R	AAGTTCTGTTCCAGGGGCCCGAATTCgagccgttcccttcggccgtcacta	RuiBiotech
Ca_V1.2-N1179W-F	tcatgatgTGGatcttcgtgggcttcgtc	Tsingke
Ca_V1.2-N1179W-R	cacgaagatCCAcatcatgaagaaggcgatg	Tsingke
Ca_V1.2-N741W-F	catctgtggaTGGtatatcctactgaatgtgttcttg	Tsingke
Ca_V1.2-N741W-R	gtaggatataCCAtccacagatgaagaggatgatg	Tsingke
Ca_V1.2-T1056Y-F	cgtgattgtcTATaccctgctgcagttcatgt	Sangon Biotech
Ca_V1.2-T1056Y-R	gcagcagggtATAgacaatcacgatgttcccga	Sangon Biotech
Ca_V1.2-Q1060M-F	caccaccctgctgATGttcatgtttgcctgcatc	Sangon Biotech
Ca_V1.2-Q1060M-R	caaacatgaaCATcagcagggtggtgacaatcac	Sangon Biotech
Ca_V1.2-Q1060F-F	caccaccctgctgTTCttcatgtttgcctgcatc	Tsingke
Ca_V1.2-Q1060F-R	caaacatgaaGAAcagcagggtggtgacaatcac	Tsingke

Supplementary Reference

1 Lohn, M. *et al.* Cilnidipine is a novel slow-acting blocker of vascular L-type calcium channels that does not target protein kinase C. *J Hypertens* **20**, 885-893 (2002). https://doi.org:10.1097/00004872-200205000-00023

2 Furukawa, T. *et al.* Five different profiles of dihydropyridines in blocking T-type Ca(2+) channel subtypes (Ca(v)3.1 (alpha(1G)), Ca(v)3.2 (alpha(1H)), and Ca(v)3.3 (alpha(1I))) expressed in Xenopus oocytes.

3 Teleb, M. *et al.* Design, synthesis and pharmacological evaluation of some substituted dihydropyrimidines with L-/T-type calcium channel blocking activities.

4 Kumar, P. P. *et al.* Synthesis and evaluation of a new class of nifedipine analogs with T-type calcium channel blocking activity.

5 Bladen, C., Gündüz Mg Fau - Şimşek, R., Şimşek R Fau - Şafak, C., Şafak C Fau - Zamponi, G. W. & Zamponi, G. W. Synthesis and evaluation of 1,4-dihydropyridine derivatives with calcium channel blocking activity.

6 Bladen, C. *et al.* 1,4-Dihydropyridine derivatives with T-type calcium channel blocking activity attenuate inflammatory and neuropathic pain.

7 Akman, D. *et al.* Focusing on C-4 position of Hantzsch 1,4-dihydropyridines: Molecular modifications, enantioseparation, and binding mechanism to L- and T-type calcium channels.
