# Supplementary material for: Structural bases of inhibitory mechanism of CaV1.2 channel inhibitors
Source: Nat Commun. 2024 Mar 30;15:2772. doi: 10.1038/s41467-024-47116-8 (PMC10981686; doi:10.1038/s41467-024-47116-8)
Supplement: Supplementary file 3 — Description of Additional Supplementary Files [file 41467_2024_47116_MOESM3_ESM.pdf]

**Description of Additional Supplementary Files**

**File Name:** Supplementary Data 1

**Description:** Diseases associated mutations of CaV1.2
