# Supplementary material for: Structural bases of inhibitory mechanism of CaV1.2 channel inhibitors
Source: Nat Commun. 2024 Mar 30;15:2772. doi: 10.1038/s41467-024-47116-8 (PMC10981686; doi:10.1038/s41467-024-47116-8)
Supplement: Supplementary file 4 — Supplementary Data 1 [file 41467_2024_47116_MOESM4_ESM.pdf]

Supplementary Data file for

Structural Bases of Inhibitory Mechanism of $Ca_V1.2$ Channel Inhibitors

Yiqing Wei, Zhuoya Yu, Lili Wang, Xiaojing Li, Na Li, Qinru Bai, Yuhang Wang, Renjie Li, Yufei Meng, Hao Xu, Xianping Wang, Yanli Dong, Zhuo Huang, Xuejun Cai Zhang, Yan Zhao

This file contains Supplementary Data.

Supplementary Data. Diseases associated mutations of $Ca_V1.2$

mutations	associated disease	structural position	site on reference	Reference
A28T	LQTS	N-terminus	A28T	1
A34V	Sudden cardiac death	N-terminus	A34V	2
A36V	schizophrenia	N-terminus	A36V	3
G37R	BrS, IVF, ERS	N-terminus	G37R	4,5
A39V	BrS	N-terminus	A39V	5
I56N	LQTS	N-terminus	I56N	6
A71V	Familial atrial fibrillation	N-terminus	A71V	7
T171M	Sudden unexplained death	DI S2	T171M	8
N300D	BrS	DI S5-P1 ECL	N300D	9
P381S	LQTS	DI S6	P381S	10
V396L	LQTS	DI S6	V396L	11
G402S	TS, LQTS	DI S6	G402S	12,13,14
G402S	LQTS	DI S6	G402S	15
S405R	TS	DI S6	S405R	16
G406R	TS	DI S6	G406R	12,17
E407A	TS	DI S6	E407A	18
M456I	LQTS	DI - DII	M456I	10
G490R	BrS/SQT	DI - DII	G490R	5
R511Q	LQTS	DII S0	R511Q	19
R518H	LQTS	DII S0	R518H	20,15
R518C	TS	DII S0	R518C	20
A519S	Sudden unexpected death	DII S0	A519S	21
N547S	BrS	DII S1-S2	N547S	22
A582D	LQTS	DII S2-S3 Amphiphilic helix	A582D	10
R590S	BrS	DII S3	R590S	2
R590C	Sudden unexplained death	DII S3	R590C	23
V596M	Sick sinus syndrome	DII S3	V596M	24
R632R	BrS	DII S4	R632R	22
S643F	TS	DII S5	S643F	25
S709N	BrS	DII S5-S6(P2)	S709N	4
C730S	LQTS	DII S6	C730S	11

带格式表格[WeiYQ [2]]

删除[WeiYQ [2]]: ，

删除[WeiYQ [2]]:

A759V	LQTS	DII S6	A759V	11
L762F	LQTS	DII S6	L762F	26
E768del	LQTS	DII S6	E768del	27
E771G	Hypertrophic cardiomyopathy	DII S6	E771G	28
S799Y	LQTS	DII - DIII	S799Y	29
K800T	High-Functioning Autism, Affective Disorder, Severe Dental Enamel Defects, Short QT Interval	DII - DIII	K800T	30
P817S	BrS, ERS	DII - DIII	P817S	4,31
K834E	LQTS	DII - DIII	K834E	32
E850 del	ERS	DII - DIII	E850 del	5
P857R	LQTS	DII - DIII	P857R	32
P857L	LQTS	DII - DIII	P857L	32
R858H	LQTS	DII - DIII	R858H	10
R860G	LQTS	DII - DIII	R860G	1
R860Q	LQTS	DII - DIII	R860Q	33
R860P	LQTS	DII - DIII	R860P	15
R1024G	TS	DIII S4	R1024G	34
C1041R	TS	DIII S5 Amphiphilic helix	C1021R	16
E1135K	BrS idiopathic QT prolongation, bradycardia, autism spectrum disorder	DIII S5-S6(P1-P2)	E1115K	5,35
R1159H	Autism	DIII S6	R1139H	36
A1174V	LQTS	DIII S6	A1154V	15
V1182L	Autism	DIII S6	V1162L	37
I1186V	LQTS	DIII S6	I1166V	1
I1186T	TS	DIII S6	I1166T	38
Q1195Term	Schizophrenia	DIII - DIV	Q1175Term	39
K1231E	TS	DIV S0	K1211E	16
V1411M	neonatal onset epileptic encephalopathy	DIV S5	V1363M	40
F1499S	restrictive cardiomyopathy	DIV S6	F1499S	41
F1513L	Sudden unexplained	DIV S6	F1465L	42

	nocturnal death syndrome			
A1521G	TS	DIV S6	A1473G	[43]
I1523M	LQTS	DIV S6	I1475M	[1]
E1544K	LQTS	CTD	E1496K	[1]
R1570Q	Autism	CTD	R1522Q	[44]
K1639T	LQTS	C-terminus	K1580T	[45]
A1765G	BrS	C-terminus	A1717G	[5]
A1781V	LQTS	C-terminus	A1733V	[46]
V1755I	stillbirth	C-terminus	V1755I	[47]
R1825C	sudden unexplained nocturnal death	C-terminus	R1777C	[48]
R1828H	BrS	C-terminus	R1780H	[22]
A1830T	Sick sinus syndrome	C-terminus	A1782T	[24]
G1831C	LQTS	C-terminus	G1783C	[10]
T1835M	BrS	C-terminus	T1787M	[5]
P1845L	stillbirth	C-terminus	P1845L	[47]
E1912-Q1916 duplication	BrS/SQT	C-terminus	E1829_Q1833 duplication	[5]
Q1916R	sudden unexplained death syndrome	C-terminus	Q1916R	[49]
R1963Q	BrS	C-terminus	R1880Q	[5]
R1989Q	LQTS	C-terminus	R1906Q	[32]
R1993Q	BrS	C-terminus	R1910Q	[22]
R1994Q	Cardiac arrhythmia	C-terminus	G1911R	[50]
R2020C	sudden cardiac death	C-terminus	R1937C	[51]
S2044N	LQTS	C-terminus	S1961N	[52]
R2056P	Cardiomyopathy with early repolarisation, SQTS	C-terminus	R1973P	[53]
R2056Q	BrS	C-terminus	R1973Q	[5]
R2056Q	stillbirth	C-terminus	R2021Q	[47]
V2097I	BrS<br>Familial atrial fibrillation	C-terminus	V2014I	[5,7]
S2103N	Sudden cardiac death	C-terminus	S2020N	[2]
N2174S	Sudden Unexplained Death	C-terminus	N2091S	[54]
D2213N	BrS	C-terminus	D2130N	[5]

The background color of pathogenic mutations in structure resolved regions are consistent

with color of $Ca_V1.2$ structure in other figures (Domain I (D_I), deep green; D_{II}, light green; D_{III}, blue; D_{IV}, mauve; C-terminal domain (CTD), orange).

Abbreviations. BrS: Brugada Syndrome, ERS: Early Repolarization Syndrome, IVF: Idiopathic Ventricular Fibrillation, LQTS: Long QT Syndrome, SQTS: Short QT Syndrome, TS:Timothy Syndrome, BrS/SQT = Brugada syndrome with shorter than normal QT.

Reference

1 Wemhöner, K. *et al.* Gain-of-function mutations in the calcium channel CACNA1C (Cav1.2) cause non-syndromic long-QT but not Timothy syndrome. *Journal of Molecular and Cellular Cardiology* **80**, 186-195, doi:10.1016/j.yjmcc.2015.01.002 (2015).

2 Brion, M. *et al.* Next generation sequencing challenges in the analysis of cardiac sudden death due to arrhythmogenic disorders. *ELECTROPHORESIS* **35**, 3111-3116, doi:https://doi.org/10.1002/elps.201400148 (2014).

3 Wang, C. *et al.* Identification of ultra-rare disruptive variants in voltage-gated calcium channel-encoding genes in Japanese samples of schizophrenia and autism spectrum disorder. *Translational Psychiatry* **12**, doi:10.1038/s41398-022-01851-y (2022).

4 Nunn, L. M. *et al.* Diagnostic yield of molecular autopsy in patients with sudden arrhythmic death syndrome using targeted exome sequencing. *EP Europace* **18**, 888-896, doi:10.1093/europace/euv285 (2015).

5 Burashnikov, E. *et al.* Mutations in the cardiac L-type calcium channel associated with inherited J-wave syndromes and sudden cardiac death. *Heart Rhythm* **7**, 1872-1882, doi:https://doi.org/10.1016/j.hrthm.2010.08.026 (2010).

6 Akgun-Dogan, O. *et al.* Mutational spectrum of congenital long QT syndrome in Turkey; identification of 12 novel mutations across KCNQ1, KCNH2, SCN5A, KCNJ2, CACNA1C, and CALM1. *Journal of Cardiovascular Electrophysiology* **33**, 262-273, doi:https://doi.org/10.1111/jce.15306 (2022).

7 Maltese, P. E. *et al.* Putative role of Brugada syndrome genes in familial atrial fibrillation.

8 Narula, N., Tester, D. J., Paulmichl, A., Maleszewski, J. J. & Ackerman, M. J. Post-mortem Whole Exome Sequencing with Gene-Specific Analysis for Autopsy-Negative Sudden Unexplained Death in the Young: A Case Series. *Pediatric Cardiology* **36**, 768-778, doi:10.1007/s00246-014-1082-4 (2014).

9 Béziau, D. M. *et al.* Complex Brugada syndrome inheritance in a family harbouring compound SCN5A and CACNA1C mutations. *Basic Res Cardiol* **109**, 446, doi:10.1007/s00395-014-0446-5 (2014).

10 Fukuyama, M. *et al.* Long QT syndrome type 8: novel CACNA1C mutations causing QT prolongation and variant phenotypes.

11 Tse, G. *et al.* Territory-Wide Chinese Cohort of Long QT Syndrome: Random Survival Forest and Cox Analyses.

12 Splawski, I. *et al.* Severe arrhythmia disorder caused by cardiac L-type calcium channel mutations.

13 Fröhler, S. *et al.* Exome sequencing helped the fine diagnosis of two siblings afflicted with atypical Timothy syndrome (TS2). *BMC Medical Genetics* **15**, 48, doi:10.1186/1471-2350-15-48 (2014).

14 Hiippala, A., Tallila J Fau - Myllykangas, S., Myllykangas S Fau - Koskenvuo, J. W., Koskenvuo Jw Fau - Alastalo, T.-P. & Alastalo, T. P. Expanding the phenotype of Timothy syndrome type 2: an adolescent with ventricular fibrillation but normal development.

15 Mellor, G. J. *et al.* Type 8 long QT syndrome: pathogenic variants in CACNA1C-encoded Cav1.2 cluster in STAC protein binding site.

16 Dufendach, K. A. *et al.* Clinical Outcomes and Modes of Death in Timothy Syndrome: A Multicenter International Study of a Rare Disorder.

17 Dufendach, K. A., Giudicessi Jr Fau - Boczek, N. J., Boczek Nj Fau - Ackerman, M. J. & Ackerman, M. J. Maternal mosaicism confounds the neonatal diagnosis of type 1 Timothy syndrome.

18 Colson, C. *et al.* Unusual clinical description of adult with Timothy syndrome, carrier of a new heterozygote mutation of CACNA1C.

19 Nakajima, T. A.-O. *et al.* Novel CACNA1C R511Q mutation, located in domain Ⅰ - Ⅱ linker, causes non-syndromic type-8 long QT syndrome.

20 Hennessey, J. A. *et al.* A CACNA1C variant associated with reduced voltage-dependent inactivation, increased CaV1. 2 channel window current, and arrhythmogenesis. *PLoS One* **9**, e106982 (2014).

21 Suktitipat, B. *et al.* Molecular investigation by whole exome sequencing revealed a high proportion of pathogenic variants among Thai victims of sudden unexpected death syndrome.

22 Fukuyama, M. *et al.* L-type calcium channel mutations in Japanese patients with inherited arrhythmias.

23 Lin, Y. *et al.* Applying High-Resolution Variant Classification to Cardiac Arrhythmogenic Gene Testing in a Demographically Diverse Cohort of Sudden Unexplained Deaths. LID - e001839 [pii] LID - 10.1161/CIRCGENETICS.117.001839 [doi].

24 Zhu, Y. B., Luo, J. W., Jiang, F. & Liu, G. Genetic analysis of sick sinus syndrome in a family harboring compound CACNA1C and TTN mutations.

25 Ozawa, J. *et al.* A novel CACNA1C mutation identified in a patient with Timothy syndrome without syndactyly exerts both marked loss- and gain-of-function effects.

26 Landstrom, A. P. *et al.* Novel long QT syndrome-associated missense mutation, L762F, in CACNA1C-encoded L-type calcium channel imparts a slower inactivation tau and increased sustained and window current.

27 Chang, S. L., Chang, C. T., Hung, W. T. & Chen, L. K. A case of congenital long QT syndrome, type 8, undergoing laparoscopic hysterectomy with general anesthesia.

28 D'Argenio, V. *et al.* DNA sequence capture and next-generation sequencing for the molecular diagnosis of genetic cardiomyopathies.

29 Burns, C. *et al.* Clinical and genetic features of Australian families with long QT syndrome: A registry-based study.

30 Endres, D. *et al.* New Cav1.2 Channelopathy with High-Functioning Autism, Affective Disorder, Severe Dental Enamel Defects, a Short QT Interval, and a Novel CACNA1C Loss-Of-Function Mutation. LID - 10.3390/ijms21228611 [doi] LID - 8611.

31 Boczek, N. J. *et al.* Identification and Functional Characterization of a Novel CACNA1C-Mediated Cardiac Disorder Characterized by Prolonged QT Intervals With Hypertrophic Cardiomyopathy, Congenital Heart Defects, and Sudden Cardiac Death. *Circ Arrhythm Electrophysiol* **8**, 1122-1132, doi:10.1161/circep.115.002745 (2015).

32 Boczek, N. J. *et al.* Exome sequencing and systems biology converge to identify novel mutations in the L-type calcium channel, CACNA1C, linked to autosomal dominant long QT syndrome.

33 Mellor G Fau - Laksman, Z. W. M. *et al.* Genetic Testing in the Evaluation of Unexplained Cardiac Arrest: From the CASPER (Cardiac Arrest Survivors With Preserved Ejection Fraction Registry). LID - e001686 [pii] LID - 10.1161/CIRCGENETICS.116.001686 [doi].

34 Kosaki, R. A.-O., Ono, H., Terashima, H. & Kosaki, K. Timothy syndrome-like condition with syndactyly but without prolongation of the QT interval.

35 Ye, D., Tester, D. J., Zhou, W., Papagiannis, J. & Ackerman, M. J. A pore-localizing CACNA1C-E1115K missense mutation, identified in a patient with idiopathic QT prolongation, bradycardia, and autism spectrum disorder, converts the L-type calcium channel into a hybrid nonselective monovalent cation channel. *Heart Rhythm* **16**, 270-278, doi:https://doi.org/10.1016/j.hrthm.2018.08.030 (2019).

36 D'Gama, A. M. *et al.* Targeted DNA Sequencing from Autism Spectrum Disorder Brains Implicates Multiple Genetic Mechanisms.

37 Iossifov, I. *et al.* The contribution of de novo coding mutations to autism spectrum disorder. doi:D - NLM: HHMIMS659757 EDAT- 2014/11/05 06:00 MHDA- 2014/12/17 06:00 CRDT- 2014/11/04 06:00 PHST- 2014/07/04 00:00 [received] PHST- 2014/10/03 00:00 [accepted] PHST- 2014/11/04 06:00 [entrez] PHST- 2014/11/05 06:00 [pubmed] PHST- 2014/12/17 06:00 [medline] AID - nature13908 [pii] AID - 10.1038/nature13908 [doi] PST - ppublish.

38 Boczek, N. J. *et al.* Novel Timothy syndrome mutation leading to increase in CACNA1C window current.

39 Purcell, S. M. *et al.* A polygenic burden of rare disruptive mutations in schizophrenia.

40 Bozarth, X. A.-O. *et al.* Expanding clinical phenotype in CACNA1C related disorders: From neonatal onset severe epileptic encephalopathy to late-onset epilepsy.

41 Kostareva, A. *et al.* Genetic Spectrum of Idiopathic Restrictive Cardiomyopathy Uncovered by Next-Generation Sequencing.

42 Huang, L. *et al.* Critical Roles of Xirp Proteins in Cardiac Conduction and Their Rare Variants Identified in Sudden Unexplained Nocturnal Death Syndrome and Brugada Syndrome in Chinese Han Population. LID - 10.1161/JAHA.117.006320 [doi] LID - e006320.

43 Gillis, J. *et al.* Long QT, syndactyly, joint contractures, stroke and novel CACNA1C mutation: Expanding the spectrum of Timothy syndrome. *American Journal of Medical Genetics Part A* **158A**, 182-187, doi:https://doi.org/10.1002/ajmg.a.34355 (2012).

44 Jiang, Y.-h. *et al.* Detection of Clinically Relevant Genetic Variants in Autism Spectrum Disorder by Whole-Genome Sequencing. *The American Journal of Human Genetics* **93**, 249-263, doi:https://doi.org/10.1016/j.ajhg.2013.06.012 (2013).

45 Kojima, A. *et al.* Refractory ventricular fibrillations after surgical repair of atrial septal defects in a patient with CACNA1C gene mutation - case report. *J Cardiothorac Surg* **12**, 118, doi:10.1186/s13019-017-0683-4 (2017).

46 Ramirez, A. H. *et al.* Novel rare variants in congenital cardiac arrhythmia genes are frequent in drug-induced torsades de pointes.

47 Munroe, P. B. *et al.* Postmortem Genetic Testing for Cardiac Ion Channelopathies in Stillbirths.

48 Zhang, L. *et al.* Does Sudden Unexplained Nocturnal Death Syndrome Remain the Autopsy-Negative Disorder: A Gross, Microscopic, and Molecular Autopsy Investigation in Southern China.

49 Hata, Y. *et al.* Postmortem genetic analysis of sudden unexplained death syndrome under 50 years of age: A next-generation sequencing study.

50 Hennessey, J. A. *et al.* A CACNA1C variant associated with reduced voltage-dependent inactivation, increased CaV1.2 channel window current, and arrhythmogenesis.

51 Campuzano, O. *et al.* Post-mortem genetic analysis in juvenile cases of sudden cardiac death. *Forensic Science International* **245**, 30-37, doi:https://doi.org/10.1016/j.forsciint.2014.10.004 (2014).

52 Nieto-Marín, P. *et al.* Digenic Heterozigosity in SCN5A and CACNA1C Explains the Variable Expressivity of the Long QT Phenotype in a Spanish Family.

53 Chen, Y. *et al.* Erratum to: Novel trigenic CACNA1C/DES/MYPN mutations in a family of hypertrophic cardiomyopathy with early repolarization and short QT syndrome.

54 Sutphin, B. S. *et al.* Molecular and Functional Characterization of Rare CACNA1C Variants in Sudden Unexplained Death in the Young.
