# Supplementary figures and images for: Structural bases of inhibitory mechanism of CaV1.2 channel inhibitors
Source: Nat Commun. 2024 Mar 30;15:2772. doi: 10.1038/s41467-024-47116-8 (PMC10981686; doi:10.1038/s41467-024-47116-8)

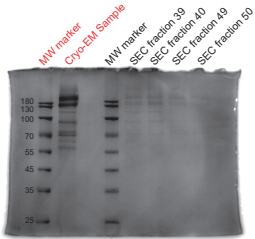

Supplement: Supplementary file 6 — Source Data [file 41467_2024_47116_MOESM6_ESM.zip › Source_Data_Files_submit/Source_Data_4-Supplementary_Figure_1c.pdf]
